# Comparative Genomic Analysis of East Asian and Non-Asian *Helicobacter pylori* Strains Identifies Rapidly Evolving Genes

**DOI:** 10.1371/journal.pone.0055120

**Published:** 2013-01-31

**Authors:** Stacy S. Duncan, Pieter L. Valk, Mark S. McClain, Carrie L. Shaffer, Jason A. Metcalf, Seth R. Bordenstein, Timothy L. Cover

**Affiliations:** 1 Department of Medicine, Vanderbilt University School of Medicine, Nashville, Tennessee, United States of America; 2 Department of Pathology, Microbiology and Immunology, Vanderbilt University School of Medicine, Nashville, Tennessee, United States of America; 3 Department of Biological Sciences, Vanderbilt University, Nashville, Tennessee, United States of America; 4 Veterans Affairs Tennessee Valley Healthcare System, Nashville, Tennessee, United States of America; Charité-University Medicine Berlin, Germany

## Abstract

*Helicobacter pylori* infection is a risk factor for the development of gastric adenocarcinoma, a disease that has a high incidence in East Asia. Genes that are highly divergent in East Asian *H. pylori* strains compared to non-Asian strains are predicted to encode proteins that differ in functional activity and could represent novel determinants of virulence. To identify such proteins, we undertook a comparative analysis of sixteen *H. pylori* genomes, selected equally from strains classified as East Asian or non-Asian. As expected, the deduced sequences of two known virulence determinants (CagA and VacA) are highly divergent, with 77% and 87% mean amino acid sequence identities between East Asian and non-Asian groups, respectively. In total, we identified 57 protein sequences that are highly divergent between East Asian and non-Asian strains, but relatively conserved within East Asian strains. The most highly represented functional groups are hypothetical proteins, cell envelope proteins and proteins involved in DNA metabolism. Among the divergent genes with known or predicted functions, population genetic analyses indicate that 86% exhibit evidence of positive selection. McDonald-Kreitman tests further indicate that about one third of these highly divergent genes, including *cagA* and *vacA*, are under diversifying selection. We conclude that, similar to *cagA* and *vacA*, most of the divergent genes identified in this study evolved under positive selection, and represent candidate factors that may account for the disproportionately high incidence of gastric cancer associated with East Asian *H. pylori* strains. Moreover, these divergent genes represent robust biomarkers that can be used to differentiate East Asian and non-Asian *H. pylori* strains.

## Introduction

Over half of the world’s human population is persistently colonized with *Helicobacter pylori,* a Gram-negative bacterium that inhabits the human stomach. *H. pylori* infection is an important risk factor for gastric adenocarcinoma, peptic ulcer disease, and gastric mucosa-associated lymphoid tissue (MALT) lymphoma [1], [2], [3], [4]. Gastric adenocarcinoma is the second leading cause of cancer-related death worldwide [5], [6], [7], [8], [9]. The incidence of this malignancy varies globally, and is particularly high in several parts of East Asia [5], [7], [8].

There is a high level of genetic diversity among *H. pylori* strains from unrelated humans, which has been attributed to an elevated mutation rate and a high rate of intraspecies genetic recombination [10], [11], [12]. Multiple populations and subpopulations of *H. pylori* with distinct geographic distributions have been recognized, based on multilocus sequence typing (MLST) analysis of conserved housekeeping genes [13], [14], [15]. Genetic diversity in *H. pylori* decreases with geographic distance from eastern Africa, a finding that is consistent with an African origin of *H. pylori* [16], [17], [18].

Considerable effort has been devoted to analyzing two important *H. pylori* virulence factors, CagA and VacA, which each exhibit a high level of intraspecies genetic diversity. VacA is a secreted pore-forming toxin that causes multiple alterations in human cells, including cell vacuolation, apoptosis, and inhibition of T-cell activation and proliferation [19]. CagA alters numerous signaling pathways, many of which are associated with malignant transformation of cells [20], [21]. Thus, CagA has been termed a “bacterial oncoprotein” [20], [22]. Within gastric epithelial cells, CagA undergoes tyrosine-phosphorylation at sites known as EPIYA motifs (EPIYA-A, B, C and D), and such phosphorylation is required for many of its actions on host cells [20], [21].

The *cagA* gene is located within a ∼40 kb chromosomal region known as the *cag* pathogenicity island (*cag* PAI), which also contains genes encoding components of a type IV secretion system that translocates CagA into gastric epithelial cells [23], [24]. Some *H. pylori* genomes contain an intact *cag* PAI, some strains contain a partial *cag* PAI, and others lack the *cag* PAI [23]. All strains contain *vacA*, but there is variation among strains in levels of *vacA* expression and VacA activity [19]. Based on observed similarities in the phylogenies of VacA and CagA in large numbers of strains, it has been suggested that these functionally interacting proteins have co-evolved in a manner that facilitates *H. pylori* colonization of the human stomach [18], [25]. Strains containing the *cag* PAI and expressing active forms of VacA are associated with a higher risk of gastric disease than are strains that lack these features [26], [27], [28].

To account for the high incidence of gastric cancer in East Asia, one hypothesis is that *H. pylori* strains from East Asia are more virulent or more frequently produce specific oncogenic factors than do strains from other parts of the world with lower rates of gastric cancer. Several lines of evidence support this hypothesis. Specifically, most *H. pylori* strains from East Asia contain the *cag* PAI and produce active forms of VacA [29]. Moreover, the CagA and VacA sequences found in many East Asian *H. pylori* strains are phylogenetically distinct from corresponding sequences found in non-Asian strains [18], [25]. East Asian strains typically produce a form of CagA that contains a tyrosine phosphorylation motif known as EPIYA-D, whereas non-Asian forms of CagA typically contain an EPIYA-C tyrosine phosphorylation motif [20], [21]. CagA proteins that contain an EPIYA-D motif have been associated with increased activity *in vitro* compared to other forms of CagA [30], [31]. East Asian strains of *H. pylori* often contain *vacA* alleles with a distinct set of 5′ polymorphisms known as type s1c and a form of the *vacA* mid-region that is highly divergent compared to *vacA* mid-regions found in non-East Asian strains [18], [32], [33]. Sequence differences in AlpA/B adhesins of East Asian strains compared to non-Asian strains have been associated with differences in functional activity, including variations in intracellular signaling [34]. The results of several studies suggest there may be many other functionally important differences when comparing East Asian and non-Asian strains [35], [36], [37]. Although several previous studies have analyzed diversity in East Asian strains of *H. pylori* compared to non-Asian strains, most of these studies were limited by the availability of only a small number of whole genome sequences or inclusion of a restricted number of genes in the analysis.

To identify candidate genes that could underlie the disproportionately high incidence of gastric cancer associated with East Asian strains, we set out to systematically compare East Asian *H. pylori* genomes with non-Asian genomes and identify rapidly evolving genes. By performing comparative genomic and phylogenetic analyses of 16 whole genome sequences (from eight East Asian and eight non-Asian *H. pylori* strains), we report the following key results: (i) 57 proteins, including CagA and VacA, are highly divergent in East Asian *H. pylori* strains compared to non-Asian strains, but relatively conserved within East Asian strains. (ii) The most highly represented functional groups of divergent proteins are hypothetical proteins, cell envelope proteins and proteins involved in DNA metabolism. (iii) These highly divergent genes exhibit significantly higher Ka/Ks ratios than control housekeeping genes, suggesting that the highly divergent genes experience more positive selection. (iv) Finally, diversifying selection has driven the divergence of about one third of the highly divergent genes, including *cagA* and *vacA*, and these genes exhibit sequence signatures of a reduction in effective population size, as measured by the mean nucleotide diversity of synonymous sites (π_s_). We propose that, similar to CagA and VacA, these proteins represent a panel of candidates that may contribute to *H. pylori* virulence and may account for the high incidence of gastric cancer associated with East Asian *H. pylori* strains. Moreover, these divergent genes represent robust biomarkers that can be used to differentiate East Asian and non-Asian *H. pylori* strains.

## Materials and Methods

### Selection of *H. pylori* Strains for Comparative Analysis

To identify strains for inclusion in this study, we evaluated all complete or nearly complete genome sequences that were available in Genbank at the time when the study was initiated. To assign these *H. pylori* strains to previously described populations and subpopulations, we used multilocus sequence typing (MLST) analysis [13]. Partial nucleotide sequences of 7 conserved housekeeping genes (*atpA*, *efp*, *mutY*, *ppa*, *trpC, yphC,* and *ureI*) from each strain were concatenated and aligned to corresponding loci from 445 reference strains contained in a MLST database (http://pubmlst.org/helicobacter) using the Muscle algorithm within MEGA5 [38]. Phylogenetic relationships were analyzed using MEGA5 with the Kimura 2-parameter model of nucleotide substitution, neighbor-joining clustering, and 10,000 bootstrap replicates. This led to the identification of 8 strains that were classified as East Asian (hspEAsian) (F16, F30, F32, F57, 35A, 51, 52 and 98-10) [35], [37], [39]. Six of these strains (F16, F30, F32, F57, 98-10, and 35A) were originally isolated from patients in Japan, and two were from Korea (51 and 52). We selected the same number of strains that were distantly related to East Asian strains and classified as non-Asian (either hpEurope or hpAfrica1), based on MLST analysis (26695, J99, HPAG1, G27, P12, B8, B38, 908) [40], [41], [42], [43], [44], [45], [46], [47].

### Identification and Classification of Genes Encoding Highly Divergent Proteins

Whole genome sequences were retrieved from Genbank and protein sequences were extracted using Bioperl [48]. As a first step to identify predicted gene products that are highly divergent in East Asian strains when compared to non-Asian strains, we conducted Blast Score Ratio (BSR) analysis [49]. This approach allows for comparisons among 3 strains (2 query strains against a single reference strain). To evaluate protein sequence similarity, two Blast score ratios are calculated, based on comparison of a query sequence (BS Q1 or BS Q2) to a reference sequence (BS Ref). Thus, BSR1 = (BS Q1)/(BS Ref) and BSR2 = (BS Q2)/(BS Ref). In this manner, all scores are normalized in the range of 0 to 1 [49]. For example, if a perfect match is found between a protein in the reference strain and a protein in a query strain, this corresponds to a BSR of 1.0. BSR analysis was performed using comparisons of each of the 8 non-Asian strains (used as Query strains) with the 8 East Asian strains (used as reference strains). For analysis of each East Asian strain, we selected proteins that yielded 0.4≤BSR≤0.93 in comparisons with at least 6 of the 8 non-Asian strains. The lower threshold value (BSR = 0.4) represents approximately 30% amino acid identity over approximately 30% of the peptide length, a commonly used threshold for peptide similarity [49]. The upper threshold value (BSR≤0.93) was chosen empirically in a manner so that we would detect proteins such as VacA (mean BSR = 0.93, calculated by averaging all of the BSR values resulting from all comparisons of East Asian and non-Asian strains), which although less divergent than CagA, is known to be divergent when comparing East Asian and non-Asian strains [18]. This approach, involving analysis of about 1500 proteins from each strain, led to the identification of 1140 candidate divergent proteins. Subsequently, we sought to refine this list by identifying protein sequences that were relatively conserved within the East Asian population, as might be expected if the corresponding genes arose from a process involving positive selection, and excluding genes that exhibited a very high rate of overall sequence divergence that was unrelated to geographic origin of strains. To do this, we performed comparisons among the 8 East Asian strains, and for each strain, we selected proteins that yielded BSR≥0.90 in comparisons with at least 6 other East Asian strains. This led to the identification of 159 candidate divergent proteins that were selected for further analysis.

As a complementary analytical approach, we further analyzed the predicted protein sequences encoded by the 8 East Asian and 8 non-Asian strains of *H. pylori* using nWayComp analysis, which allows for the comparison of protein sequences among multiple strains at the whole-genome level [50]. nWayComp analysis compares DNA or protein sequences, searches for homologous sequences among multiple strains, and identifies genes or proteins that are either unique to a particular strain or are encoded in multiple strains. For each set of orthologous sequences, we generated a table of maximum size n×n, where n = 16, which displayed amino acid sequence identities among the analyzed sequences. Mean percent amino acid identities were calculated based on all possible comparisons of East Asian sequences with orthologous non-Asian sequences. Sequences were excluded if there were marked differences in peptide lengths (when compared to orthologous sequences in other strains) or in cases in which proteins had been incorrectly identified as orthologues. This manual curation resulted in a reduction in the number of highly divergent gene products from 159 to 57. To further examine the divergent gene products selected with BSR and nWayComp analyses, neighbor-joining trees were constructed for each of the 57 proteins, using the program Geneious (Drummond AJ, Ashton B, Buxton S, Cheung M, Cooper A, Heled J, Kearse M, Moir R, Stones-Havas S, Sturrock S, Thierer T, Wilson A (2010) Geneious v5.1, available from http://www.geneious.com). Trees were inspected to determine whether East Asian sequences clustered together or whether East Asian and non-Asian sequences were intermingled. We also performed Bayesian analyses of a subset of trees to ensure that the neighbor joining inference methods were accurate [51], [52]. ProtTest model selection and Bayesian inference generally recapitulated the patterns observed in neighbor joining trees, and consistently revealed clustering of East Asian sequences. Predicted main functional classes and sub-functional classes for each of 57 divergent sequences were assigned based on previous classifications (J. Craig Venter Institute Comprehensive Microbial Resource database).

### Analysis of Mean Nucleotide Diversity and Positive Selection

For analysis of nucleotide diversity and positive selection, sequences of orthologous genes were aligned using Muscle in Geneious, version 5.4.5. Hypervariable regions and insertions/deletions (indels) were manually removed. Nucleotide divergence at non-synonymous and synonymous sites (Ka and Ks, respectively, with Jukes and Cantor correction) and silent site diversity (π_s_) was calculated for each set of orthologous sequences with the program DnaSP (http://www.ub.edu/dnasp). Sequences from East Asian strains were compared with corresponding sequences from non-Asian strains.

The McDonald-Kreitman test (http://mkt.uab.es/mkt/) for positive selection [53] was performed with the exclusion of low-frequency variants less than or equal to 15% to reduce artifacts associated with detecting adaptive evolution. The neutrality index (NI) was calculated as follows: NI = (Pn/Ps)/(Dn/Ds), where P is polymorphic within the population, D is divergence or fixed difference between populations, n is nonsynonymous, and s is synonymous.

## Results

### MLST Analysis of *H. pylori* Strains

**Figure 1:**
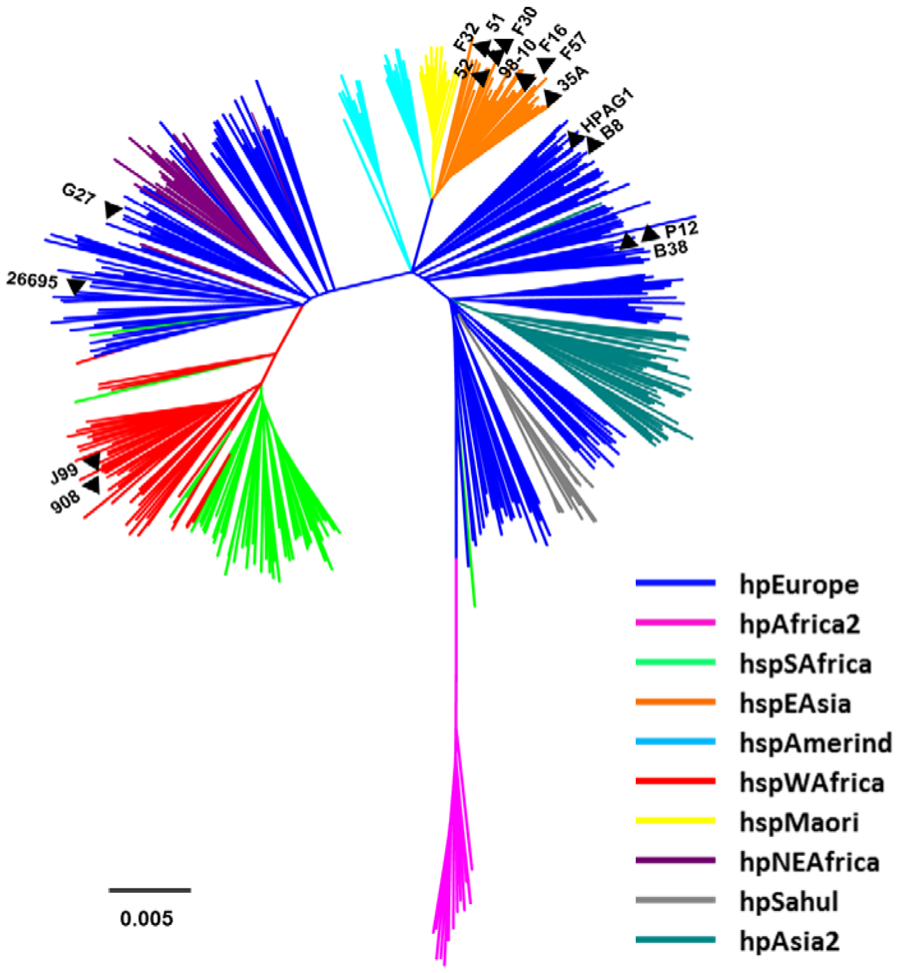
MLST analysis of *H. pylori* strains included in this study. Nucleotide sequences of 7 conserved housekeeping genes (*atpA*, *efp*, *mutY*, *ppa*, *trpC*, *ureI*, and *yphC*) from 16 strains of *H. pylori* were concatenated and compared to corresponding loci from 445 reference strains (see Methods). Eight strains (98-10, 35A, 51, 52, F16, F30, F32 and F57) were classified as hspEAsia, six strains (26695, HPAG1, G27, P12, B8 and B38) were classified as hpEurope and two strains (J99 and 908) were classified as hspWAfrica.

In an analysis of *H. pylori* strains for which complete or nearly-complete genome sequences were available in Genbank when this study was initiated, we identified eight strains that were classified as East Asian (hspEAsia), based on MLST analysis (Fig. 1). We selected the same number of genome sequences from strains that were classified as non-Asian, based on MLST analysis. Six of the latter strains were classified as hpEurope and two were classified as hspWAfrica (Fig. 1). The assignment of the 16 strains to these population groups is consistent with previous analyses [23].

### Identification of Highly Divergent Alleles in East Asian Strains

The sequences of two virulence factors, CagA and VacA, are known to be highly divergent in East Asian strains compared to non-Asian strains [18], [25]. Consistent with expectations, all eight East Asian strains in the current study (98-10, 35A, F16, F30, F32, F57, 51 and 52) encode CagA proteins with an EPIYA-D motif, whereas 7 non-Asian strains (26695, J99, HPAG1, G27, P12, B8 and 908) encode CagA with an EPIYA-C motif (Table 1). The eight non-Asian strain (B38) does not contain the *cag* pathogenicity island, and therefore, this strain does not contain *cagA*. Seven out of the eight East Asian strains contain an s1c *vacA* allele, whereas the s1c genotype was not identified in any of the non-Asian strains. These features of CagA and VacA conform to the profiles that are predicted based on the MLST classification of the 16 strains.

**Table 1:** Classification of CagA and VacA in 16 *H. pylori* strains.

Strain	*cag* PAI	CagA type (EPIYA)	VacA type
98-10	+	EPIYA-D	s1c/i1/m1
35A	+	EPIYA-D	s1c/i1/m1
F16	+	EPIYA-D	s1c/i1/m1
F30	+	EPIYA-D	s1c/i1/m1
F32	+	EPIYA-D	s1c/i1/m1
F57	+	EPIYA-D	s1a/i1/m1
51	+	EPIYA-D	s1c/i1/m1
52	+	EPIYA-D	s1c/i1/m1^a^
26695	+	EPIYA-C	s1a/i1/m1
J99	+	EPIYA-C	s1a/i1/m1
HPAG1	+	EPIYA-C	s1a/i1/m1
G27	+	EPIYA-C	s1b/i1/m1
P12	+	EPIYA-C	s1a/i1/m1
B8	+	EPIYA-C	s1a/i2/m2^a^
908	+	EPIYA-C	s1b/i1/m1
B38	−	Not applicable^b^	s2/i2/m2

a VacA is truncated in strains 52 and B8.

b The *cag* PAI is absent from strain B38.

Neighbor-joining tree analyses confirmed that CagA and VacA were highly divergent in East Asian strains of *H. pylori* compared to non-Asian strains (Fig. S1). Similar analysis of concatenated housekeeping gene sequences revealed that East Asian strains were distinguishable from non-Asian strains, but the level of divergence among housekeeping genes was much lower than observed for CagA and VacA (compare Fig. S1C with Fig. S1A, B). When comparing 7 housekeeping genes from East Asian and non-Asian strains of *H. pylori*, the mean amino acid identity (based on all possible comparisons of orthologous sequences) was 96%. In contrast, the mean amino acid identity of CagA sequences between groups was 77%, and the mean amino acid identity of VacA sequences between groups was 87%. Therefore, when East Asian strains are compared to non-Asian strains, there is a much higher level of divergence in CagA and VacA than in the products of housekeeping genes.

To identify other proteins encoded by East Asian strains that might be highly divergent compared to those encoded by non-Asian strains, we compared the protein sequences of eight East Asian *H. pylori* strains and eight non-Asian strains, using Blast Score Ratio and nWayComp analyses (described in Methods). We identified 57 predicted gene products, including CagA and VacA, that were highly divergent between East Asian and non-Asian strains and relatively conserved within the East Asian group (Table 2). Analysis of these proteins indicated that the intergroup differences in amino acid identities ranged from 71%–91% (Table 3). As shown in Table 2, the 57 divergent proteins were grouped based upon their predicted main functional class (J. Craig Venter Institute Comprehensive Microbial Resource database). The most highly represented groups were hypothetical proteins, cell envelope proteins, and proteins involved in DNA metabolism (Table 2).

**Table 2:** Proteins that are highly divergent in East Asian and non-Asian strains of *H. pylori.*

Main role^a^	Subrole	Gene ID^b^	Annotationc,d
Cell envelope	Other	HP0009	outer membrane protein HopZ (omp1)
Cell envelope	Other	HP0025	outer membrane protein HopD (omp2)
Cell envelope	Other	HP1243	outer membrane protein BabA (omp28)
Cell envelope	Other	HP0373	outer membrane protein HomC/HomD
Cell envelope	Other	NA^e^	outer membrane protein HomB
Cell envelope	Other	HP0725	outer membrane protein SabA/HopP (omp17)
Cell envelope	Other	HP0923	outer membrane protein HopK (omp12)
Cell envelope	Other	HP0229	outer membrane protein HopA (omp6)
Cell envelope	Other	HP1157	outer membrane protein HopL (omp26)
Cell envelope	Other	HP0609/0610	vacuolating cytotoxin (VacA)-like protein
Cell envelope	Other	HP0922	vacuolating cytotoxin (VacA)-like protein
Cell envelope	Other	HP0492	HpaA-like protein
Cell envelope	Biosynthesis and degradation of surface polysaccharides and lipopolysaccharides	HP0651	alpha-(1,3)-fucosyltransferase
Cell envelope	Biosynthesis and degradation of surface polysaccharides and lipopolysaccharides	HP0159	lipopolysaccharide 1,2-glucosyltransferase (RfaJ)
Cell envelope	Biosynthesis and degradation of murein sacculus and peptidoglycan	HP0160	cysteine-rich protein D/beta-lactamase HcpD
Cellular processes	Pathogenesis	HP0547	cytotoxin associated protein A (CagA)
Cellular processes	Toxin production and resistance	HP0887	vacuolating cytotoxin A (VacA)
Cellular processes	Chemotaxis and motility	HP0906	flagellar hook-length control protein
DNA metabolism	DNA replication, recombination, and repair	HP1553	recombination protein RecB/helicase
DNA metabolism	DNA replication, recombination, and repair	HP0661	ribonuclease H (RnhA)
DNA metabolism	DNA replication, recombination, and repair	HP1323	ribonuclease HII (RnhB)
DNA metabolism	Restriction/modification	HP0463	type I restriction enzyme M protein/HsdM
DNA metabolism	Restriction/modification	HP0850	type I restriction enzyme M protein (HsdM)
DNA metabolism	Restriction/modification	HP1354	type IIG restriction-modification enzyme/adenine specific DNA methyltransferase
DNA metabolism	Restriction/modification	HP1371	type III restriction enzyme R protein
Protein fate	Degradation of proteins, peptides, and glycopeptides	HP0806	metalloprotease
Protein fate	Protein and peptide secretion and trafficking	HP1255	preprotein translocase subunit SecG
Protein synthesis	tRNA and rRNA base modification	HP1415	tRNA delta(2)-isopentenylpyrophosphate transferase (MiaA)
Protein synthesis	tRNA aminoacylation	HP1513	selenocysteine synthase (SelA)/L-seryl-tRNA(Sec)selenium transferase
Purines, pyrimidines, nucleosides, and nucleotides	Purine ribonucleotide biosynthesis	HP1530	purine nucleoside phosphorylase (PunB)
Transcription	RNA processing	HP0640	poly(A) polymerase (PapS)
Unknown function	General	HP0322	poly E-rich protein
Hypothetical	Conserved	HP0728	tRNA(Ile)-lysidine synthase (TilS)
Hypothetical	Conserved	HP0729	probable ATP/GTP binding protein
Hypothetical	Conserved	HP1250	bacterial SH3 domain protein
Hypothetical	Conserved	HP0852	excinuclease ATPase subunit
Hypothetical	Conserved	HP1265	NADH-ubiquinone oxidoreductase chain F (NuoF)
Hypothetical	Conserved	HP0721	hypothetical protein
Hypothetical	Conserved	HP0636	hypothetical protein
Hypothetical	Conserved	HP1579	hypothetical protein
Hypothetical	Conserved	HP0861	hypothetical protein
Hypothetical	Conserved	HP0384	hypothetical protein
Hypothetical	Conserved	HP0635	hypothetical protein
Hypothetical	Conserved	HP0897	hypothetical protein
Hypothetical	Conserved	HP0398	hypothetical protein
Hypothetical	Conserved	HP0629	hypothetical protein
Hypothetical	Conserved	HP0973	hypothetical protein
Hypothetical	Conserved	HP0167	hypothetical protein
Hypothetical	Conserved	HP0120	hypothetical protein
Hypothetical	Conserved	HP0583	hypothetical protein
Hypothetical	Conserved	HP0119	hypothetical protein
Hypothetical	Conserved	HP0681	hypothetical protein
Hypothetical	Conserved	HP1321	hypothetical protein
Hypothetical	Conserved	HP0833	hypothetical protein
Hypothetical	Conserved	HP0338	hypothetical protein
Hypothetical	Conserved	HP0061	hypothetical protein
Hypothetical	Conserved	HP1322	hypothetical protein
**Control Group**			
Energy metabolism	ATP-proton motive force interconversion	HP1134	ATP synthase F0F1 subunit alpha (AtpA)
Protein synthesis	Translation factors	HP0177	elongation factor P (Efp)
DNA metabolism	DNA replication, recombination, and repair	HP0142	A/G-specific adenine glycosylase (MutY)
Central intermediary metabolism	Phosphorus compounds	HP0620	inorganic pyrophosphatase (Ppa)
Tryptophan biosynthesis	Aromatic amino acid family	HP1279	anthranilate isomerase (TrpC)
Central intermediary metabolism	Other	HP0071	urease accessory protein (UreI)
Unknown function	General	HP0834	GTP-binding protein (YphC)

a Assignment of genes into functional groups is based on classifications of *H. pylori* 26695 genes reported in the JCVI Comprehensive Microbial Resource database, based on analysis of three *H. pylori* genomes (26695, J99 and HPAG1).

b Gene numbers in *H. pylori* reference strain 26695 are shown.

c Annotations are based on data reported in the JCVI Comprehensive Microbial Resource database or data reported in Genbank at the time when this study was undertaken.

d Three proteins initially classified as “hypothetical” were subsequently found to exhibit similarity to proteins of known function. These include HP0861 [corresponding to heavy metal (copper tolerance) in *Shewanella* and integral membrane protein in *Campylobacter*], HP0635 (corresponding to hydrogenase E in *Campylobacter*) and HP1321 (corresponding to an ATPase in *Wolinella* and other species). Conserved domain analysis indicates that HP0861 belongs to the Dsb superfamily, HP0384 belongs to the SPOR superfamily, and HP1321 belongs to both the P-loop-containing nucleoside triphosphosphate hydrolase superfamily and the helix-turn-helix superfamily.

e Not applicable. HomB is absent from strain 26695.

**Table 3:** Analysis of nucleotide diversity.^a^

Annotation	Gene ID (26695)	Mean % aa identity (EA vs. Non-EA)^b^	π_a_-EA	π_a_-Non EA	π_s_-EA	π_s_-Non EA	K_a_/K_s_ (EA-NEA)^c^
HopZ (omp1)	HP0009	73.61	0.069	0.039	0.228	0.186	0.264
HopD (omp2)	HP0025	88.96	0.015	0.042	0.097	0.212	0.224
BabA (omp28)	HP1243	87.65	0.050	0.053	0.186	0.266	0.226
HomC/HomD	HP0373	80.21	0.012	0.068	0.073	0.272	0.293
HomB	NA^d^	86.77	0.053	0.048	0.195	0.236	0.220
SabA/HopP/(omp17)	HP0725	82.78	0.070	0.039	0.171	0.183	0.299
HopK (omp12)	HP0923	89.23	0.021	0.040	0.100	0.205	0.197
HopA (omp6)	HP0229	89.24	0.040	0.043	0.116	0.170	0.259
HopL (omp26)	HP1157	89.58	0.032	0.036	0.132	0.202	0.210
VacA-like protein	HP0609/0610	91.09	0.024	0.037	0.144	0.251	0.162
VacA-like protein	HP0922	89.93	0.020	0.027	0.083	0.167	0.197
HpaA-like protein	HP0492	71.03	0.014	0.038	0.053	0.128	0.403
alpha-(1,3)-fucosyltransferase	HP0651	81.68	0.023	0.057	0.174	0.294	0.218
lipopolysaccharide 1,2-glucosyltransferase (rfaJ)	HP0159	86.99	0.020	0.057	0.063	0.193	0.331
cysteine-rich protein D/beta-lactamase (hcpD)	HP0160	89.61	0.022	0.035	0.088	0.141	0.320
cytotoxin associated protein A (cagA)	HP0547	77.74	0.018	0.054	0.067	0.116	0.414
vacuolating cytotoxin A (vacA)	HP0887	87.39	0.013	0.057	0.097	0.203	0.260
flagellar hook-length control protein	HP0906	85.76	0.030	0.049	0.108	0.178	0.279
recombination protein RecB/helicase	HP1553	89.52	0.020	0.032	0.102	0.101	0.229
ribonuclease H (rnhA)	HP0661	79.16	0.020	0.020	0.149	0.076	0.206
ribonuclease HII (rnhB)	HP1323	87.90	0.035	0.042	0.165	0.173	0.222
type I restriction enzyme M protein (hsdM)	HP0463	89.23	0.025	0.037	0.091	0.147	0.250
type I restriction enzyme M protein (hsdM)	HP0850	87.75	0.026	0.044	0.131	0.208	0.213
type IIG restriction-modification enzyme	HP1354	82.75	0.031	0.072	0.098	0.242	0.321
type III restriction enzyme R protein	HP1371	83.71	0.031	0.036	0.093	0.155	0.264
metalloprotease	HP0806	87.69	0.020	0.047	0.090	0.231	0.251
preprotein translocase subunit secG	HP1255	89.15	0.012	0.024	0.087	0.169	0.179
tRNA delta(2)-isopentenylpyrophosphate transferase (miaA)	HP1415	80.77	0.020	0.055	0.100	0.195	0.268
selenocysteine synthase (SelA)/L-seryl-tRNA(Sec) selenium transferase	HP1513	89.50	0.024	0.038	0.093	0.195	0.221
purine nucleoside phosphorylase (punB)	HP1530	89.03	0.016	0.037	0.091	0.200	0.223
poly(A) polymerase (papS)	HP0640	89.11	0.021	0.034	0.106	0.187	0.190
poly E-rich protein	HP0322	72.27	0.027	0.048	0.105	0.189	0.243
tRNA(Ile)-lysidine synthase	HP0728	89.96	0.017	0.034	0.078	0.150	0.236
probable ATP/GTP binding protein	HP0729	88.15	0.035	0.031	0.151	0.158	0.190
bacterial SH3 domain protein	HP1250	77.58	0.038	0.056	0.122	0.131	0.445
Excinuclease ATPase subunit	HP0852	83.85	0.038	0.053	0.100	0.194	0.284
NADH-ubiquinone oxidoreductase chain F	HP1265	88.97	0.018	0.038	0.078	0.186	0.218
**Control group**							
ATP synthase F0F1 subunit alpha (atpA)	HP1134	98.00	0.003	0.003	0.076	0.105	0.027
elongation factor P (efp)	HP0177	98.00	0.002	0.003	0.107	0.159	0.020
A/G-specific adenine glycosylase (mutY)	HP0142	94.00	0.011	0.026	0.095	0.223	0.114
inorganic pyrophosphatase (ppa)	HP0620	96.00	0.004	0.005	0.060	0.123	0.092
anthranilate isomerase (trpC)	HP1279	94.00	0.020	0.032	0.088	0.188	0.173
urease accessory protein (ureI)	HP0071	97.00	0.001	0.007	0.047	0.103	0.061
GTP-binding protein (yphC)	HP0834	96.00	0.008	0.018	0.078	0.154	0.096

a Outlier sequences were not removed prior to these analyses.

b The mean % amino acid identity when comparing East Asian (EA) and non-EA sequences was significantly higher for the control group of housekeeping genes than for the group of divergent genes.

c The mean Ka/Ks ratio, calculated based on comparison of East Asian sequences with non-EA (NEA) sequences, was significantly higher for the group of divergent genes than for the control group of housekeeping genes.

d Not applicable. HomB is absent from strain 26695.

**Figure 2:**
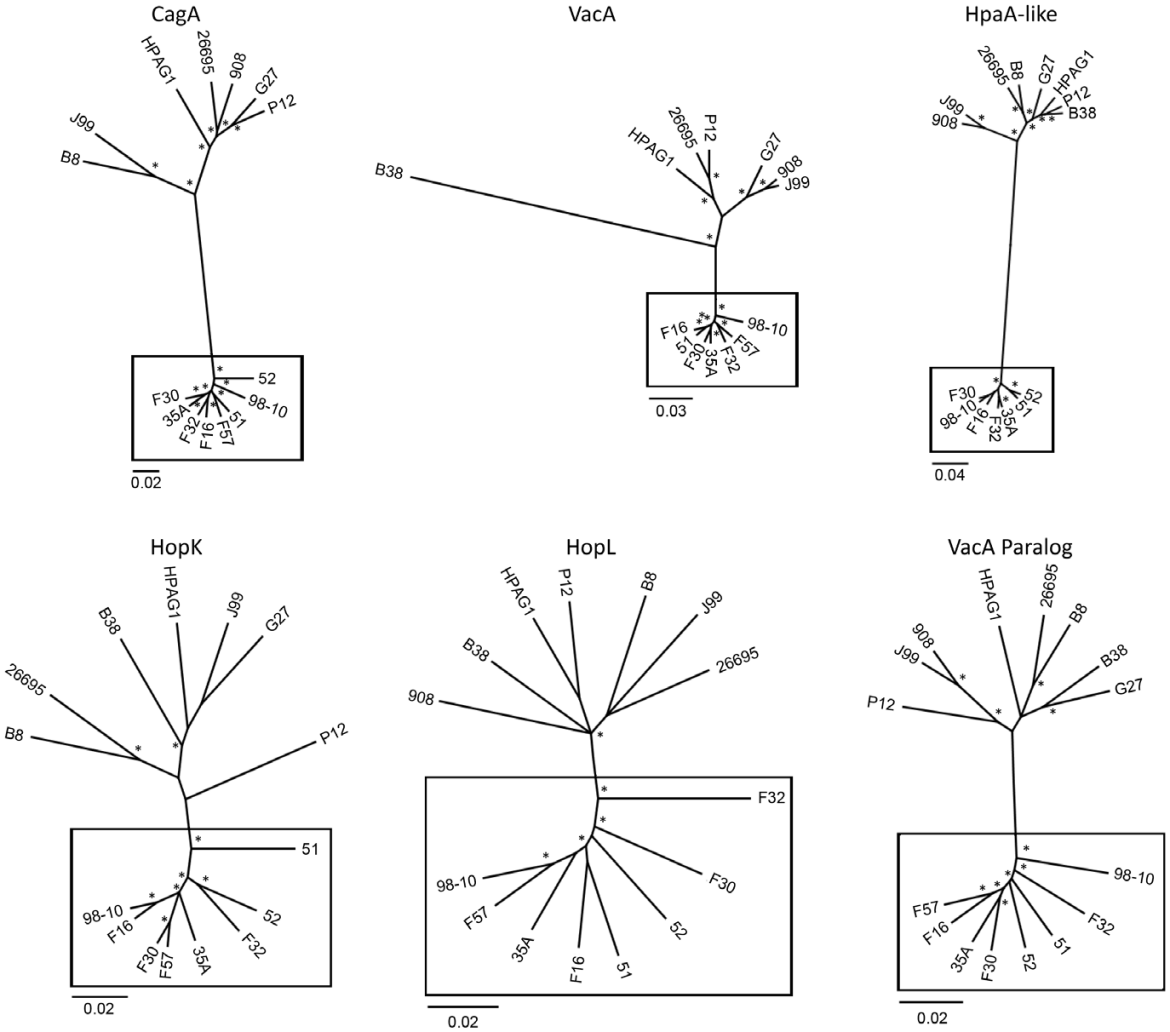
Bayesian phylogenies for six representative proteins that are highly divergent in East Asian *H. pylori* strains compared to non-Asian strains. These include CagA (HP0547), VacA (HP0887), HpaA-like protein (HP0492), HopK (HP0923), HopL (HP1157), and a VacA-like protein (HP0922). The best available model of evolution was determined with ProtTest and phylogenies were inferred using MrBayes. Asterisks indicate posterior probabilities greater than 0.75. Sequences from East Asian strains (boxed) are highly divergent when compared to corresponding amino acid sequences from non-Asian strains of *H. pylori*. Scale bars show number of substitutions per site.

In order to link predicted functions with patterns of molecular evolution, we identified 37 genes that had been assigned to predicted functional groups or for which annotations were available (Table 2), and focused further analyses on these proteins. We identified intact coding sequences for thirteen of the 37 proteins in all 16 strains, and thus, these 13 genes (HP0159, HP0160, HP0229, HP0640, HP0728, HP0806, HP0906, HP0922, HP1255, HP1265, HP1323, HP1415, HP1513) represent a subset of the core genome. The apparent absence of intact coding sequences was most commonly observed in strains 908 and 98-10. There are many possible reasons for why a particular gene sequence might not be identified in an individual strain; these include absence of the gene from the strain, presence of a truncated gene or pseudogene, or failure to detect the gene due to shortcomings in sequencing, assembly or annotation of a genome. To examine relationships among sequences for the sets of divergent gene products, we constructed neighbor-joining phylogenetic trees for each of the 37 predicted proteins. Phylogenetic analysis (prior to removal of outliers) revealed that for 17 of the 37 proteins, all of the East Asian sequences formed a well-defined cluster that was distinct from non-Asian sequences. These include CagA (HP0547), VacA (HP0887), HpaA paralog (HP0492), HopL (HP1157), a VacA-like protein (HP0922), HP0159, HP0160, HP0651, HP0728, HP0906, HP1243, HP1250, HP1255, HP1265, HP1323, HP1415, and HP1553. Representative phylogenetic trees are shown in Fig. 2. For the remaining 20 proteins, at least one sequence did not cluster within the expected East Asian or non-Asian group; these outlier sequences were randomly distributed among the 16 strains analyzed, and may have arisen through recombination. All subsequent analyses of the 37 proteins were performed both with and without the removal of outlier sequences, and the two approaches generally yielded similar results.

### Analysis of Nucleotide Diversity and Positive Selection

**Figure 3:**
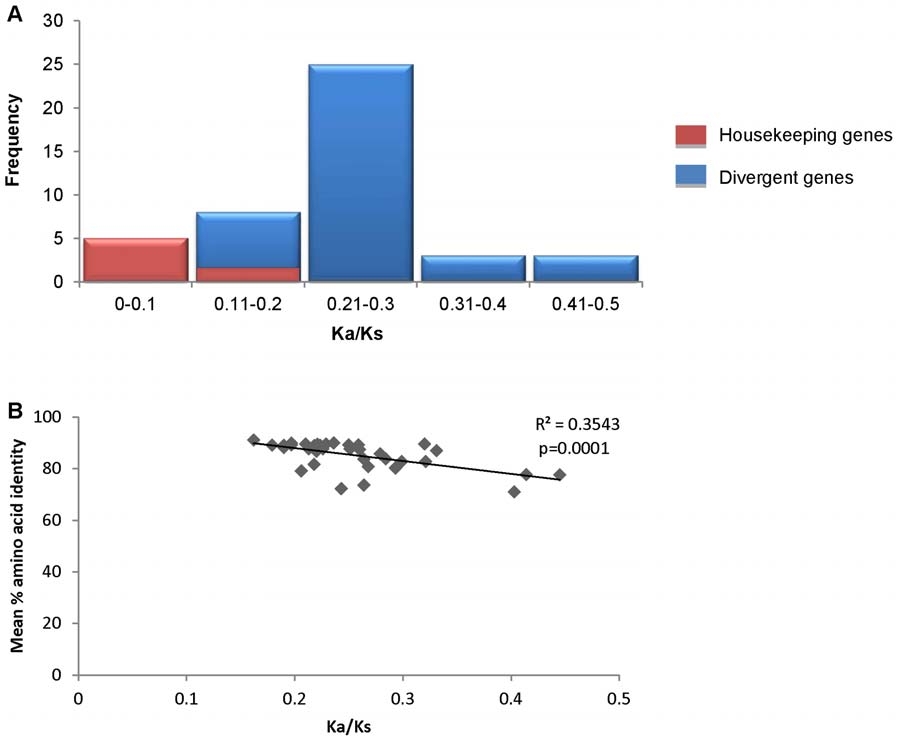
Analyses of Ka/Ks values for 37 divergent genes with predicted functions and seven housekeeping genes. Gene-wide Ka/Ks ratios were calculated, comparing sequences from East Asian strains with corresponding sequences from non-Asian strains, without the removal of outliers. (A) Distribution of Ka/Ks values. Ka/Ks values of highly divergent genes were significantly higher than Ka/Ks values of housekeeping genes. (B) Simple linear regression analysis comparing Ka/Ks values with mean % amino acid identity values (East Asian vs. non-Asian) for the 37 highly divergent proteins.

To determine if the 37 genes were under positive selection, we calculated gene-wide Ka/Ks ratios, comparing sequences from East Asian strains with corresponding sequences from non-Asian strains (Table 3). As shown in Fig. 3A, Ka/Ks values of highly divergent genes were higher than Ka/Ks values of housekeeping genes. Specifically, the mean Ka/Ks ratio for the group of divergent 37 genes (0.256+0.064) was significantly higher than that of a control group of 7 housekeeping genes (0.083+0.049) (Mann-Whitney U test, p<0.001) (Table 3 and Table S1). Therefore, as a group, the 37 divergent genes are under less purifying selection than the seven housekeeping genes, as expected. Furthermore, the Ka/Ks ratios of the 37 divergent genes were negatively correlated with the level of amino acid similarity (comparing East Asian and non-Asian sequences) (Fig. 3B). These results indicate that sequences with higher protein sequence divergence exhibit less purifying selection.

The elevated Ka/Ks ratios in the 37 divergent genes and the inverse correlation of Ka/Ks ratios with protein sequence divergence could have arisen by either increased positive selection or neutral evolution. To test which of the 37 divergent genes were under positive diversifying selection, we used the McDonald-Kreitmann test (MKT) to compare sequences from the East Asian population with sequences from the non-Asian population. The MKT analyzes the neutral theory prediction that the ratio of synonymous-to-nonsynonymous polymorphisms (Ps/Pn) within groups should be the same as the ratio of synonymous-to-nonsynonymous divergence (Ds/Dn) between groups. Among the 37 divergent genes, eight were determined to be under diversifying selection (Table 4), and this number increased to 12 once outliers were excluded (Table S2). One of the 7 housekeeping genes, *ppa,* was also determined to be under diversifying selection, but this gene exhibited a relatively low level of amino acid divergence. Excess nonsynonymous fixation, one signature of adaptive protein evolution, causes the Neutrality Index (NI) in the MKT to be less than 1. For all statistically significant MKT comparisons, the NI was <0.702 (Table 4).

**Table 4:** Analysis of positive selection using McDonald-Kreitman test.^a^

Annotation	Gene ID (26695)	*Dn*	*Ds*	*Pn*	*Ps*	*P* value	NI^b^	α-Value^c^
HopZ (omp1)	HP0009	12.07	16.44	285	346	0.765	1.122	−0.122
HopD (omp2)	HP0025	22.21	25.98	95	141	0.561	0.788	0.211
BabA (omp28)	HP1243	28.33	28.08	127	244	0.020	0.515*	0.484
HomC/HomD	HP0373	38.63	37	177	212	0.373	0.799	0.200
HomB	NA^d^	5.01	9.13	198	291	0.702	1.239	−0.239
SabA/HopP/(omp17)	HP0725	21.21	15.39	177	215	0.137	0.597	0.402
HopK (omp12)	HP0923	13.13	14.62	58	77	0.672	0.838	0.161
HopA (omp6)	HP0229	4.01	9.18	126	113	0.114	2.552	−1.552
HopL (omp26)	HP1157	29.20	24.52	201	307	0.036	0.549*	0.450
VacA-like protein	HP0609/0610	36.11	38.47	743	1321	0.028	0.599*	0.400
VacA-like protein	HP0922	58.41	57.43	325	488	0.032	0.654*	0.345
HpaA-like protein	HP0492	79.15	57.12	45	41	0.399	0.792	0.207
alpha-(1,3)-fucosyltransferase	HP0651	27.51	24.45	145	220	0.070	0.585	0.414
lipopolysaccharide 1,2-glucosyltransferase (rfaJ)	HP0159	12.11	6.10	76	71	0.232	0.539	0.460
cysteine-rich protein D/beta-lactamase HcpD	HP0160	8.06	3.03	45	63	0.047	0.268*	0.731
cytotoxin associated protein A (cagA)	HP0547	144.15	79.93	209	165	0.041	0.702*	0.297
vacuolating cytotoxin A (vacA)	HP0887	64.97	51.03	183	271	0.002	0.530*	0.469
flagellar hook-length control protein	HP0906	13.09	9.18	106	103	0.469	0.721	0.278
recombination protein RecB/helicase	HP1553	40.50	28.97	132	167	0.033	0.565*	0.434
ribonuclease H (rnhA)	HP0661	4.04	2.04	9	17	0.150	0.267	0.732
ribonuclease HII (rnhB)	HP1323	6.05	7.29	34	50	0.736	0.819	0.180
type I restriction enzyme M protein (hsdM)	HP0463	11.06	11.25	149	142	0.881	1.067	−0.067
type I restriction enzyme M protein (hsdM)	HP0850	17.16	14.43	93	122	0.242	0.641	0.358
type IIG restriction-modification enzyme	HP1354	8.02	6.04	404	365	0.738	0.834	0.165
type III restriction enzyme R protein	HP1371	10.03	13.25	228	188	0.269	1.602	−0.602
metalloprotease	HP0806	8.09	4.08	41	52	0.141	0.398	0.601
preprotein translocase subunit SecG	HP1255	5.03	4.09	23	38	0.314	0.491	0.508
tRNA delta(2)-isopentenylpyrophosphate transferase (miaA)	HP1415	9.09	9.34	55	63	0.828	0.897	0.102
selenocysteine synthase (SelA)/L-seryl-tRNA(Sec) selenium transferase	HP1513	13.13	12.44	60	94	0.237	0.604	0.395
purine nucleoside phosphorylase (punB)	HP1530	7.07	4.10	30	40	0.202	0.434	0.565
poly(A) polymerase (papS)	HP0640	7.03	10.29	52	88	0.778	0.864	0.135
poly E-rich protein	HP0322	14.15	6.12	64	62	0.111	0.446	0.553
tRNA(Ile)-lysidine synthase	HP0728	8.05	7.16	51	55	0.725	0.825	0.174
probable ATP/GTP binding protein	HP0729	10.08	21.39	90	90	0.062	2.121	−1.121
bacterial SH3 domain protein	HP1250	17.52	8.44	39	26	0.505	0.722	0.277
excinuclease ATPase subunit	HP0852	6.03	6.14	95	82	0.779	1.180	−0.180
NADH-ubiquinone oxidoreductase chain F	HP1265	7.05	3.03	54	58	0.186	0.400	0.599
**Control group**								
ATP synthase F0F1 subunit alpha (atpA)	HP1134	0	2	5	79	0.721	Null	Null
elongation factor P (efp)	HP0177	0	1	3	47	0.800	Null	Null
A/G-specific adenine glycosylase (mutY)	HP0142	1	3.03	32	81	0.023	1.197	−0.197
inorganic pyrophosphatase (ppa)	HP0620	5.04	5.16	1	22	0.001	0.046*	0.953
anthranilate isomerase (trpC)	HP1279	5.01	7.11	62	109	0.722	0.807	0.192
urease accessory protein (ureI)	HP0071	0	2.01	8	43	0.541	Null	Null
GTP-binding protein (yphC)	HP0834	2	4.06	17	50	0.680	0.689	0.310

a Outlier sequences were not removed prior to these analyses.

b The neutrality index (NI) was calculated from the ratio of the number of polymorphisms to the number of substitutions as follows: NI = (*Pn*/*Ps*)/(*D*n/*Ds*), where *P* is polymorphic within the population, *D* is divergence or fixed difference between populations, *n* is nonsynonymous, and *s* is synonymous.

c The proportion of adaptive substitutions that ranges from - ∞ to 1 and is estimated as 1 - NI.

d Not applicable. HomB is absent from strain 26695.

* Asterisks indicate genes showing signatures of diversifying selection.

Genes subject to positive, diversifying selection will exhibit a reduction in effective population size, as measured by the mean nucleotide diversity of synonymous sites (π_s_). The basic reason is that as positive selection sweeps alleles through a population, the silent site variation hitches alongside the selected region, thereby reducing π_s_ in comparison to alleles under neutral evolution. Ka/Ks ratios would then negatively correlate with π_s_. Alternatively, elevated Ka/Ks ratios may reflect an increase in neutral evolution, and in this case, the elevated Ka/Ks ratios should be associated with elevated π_s_ due to increased drift.

**Figure 4:**
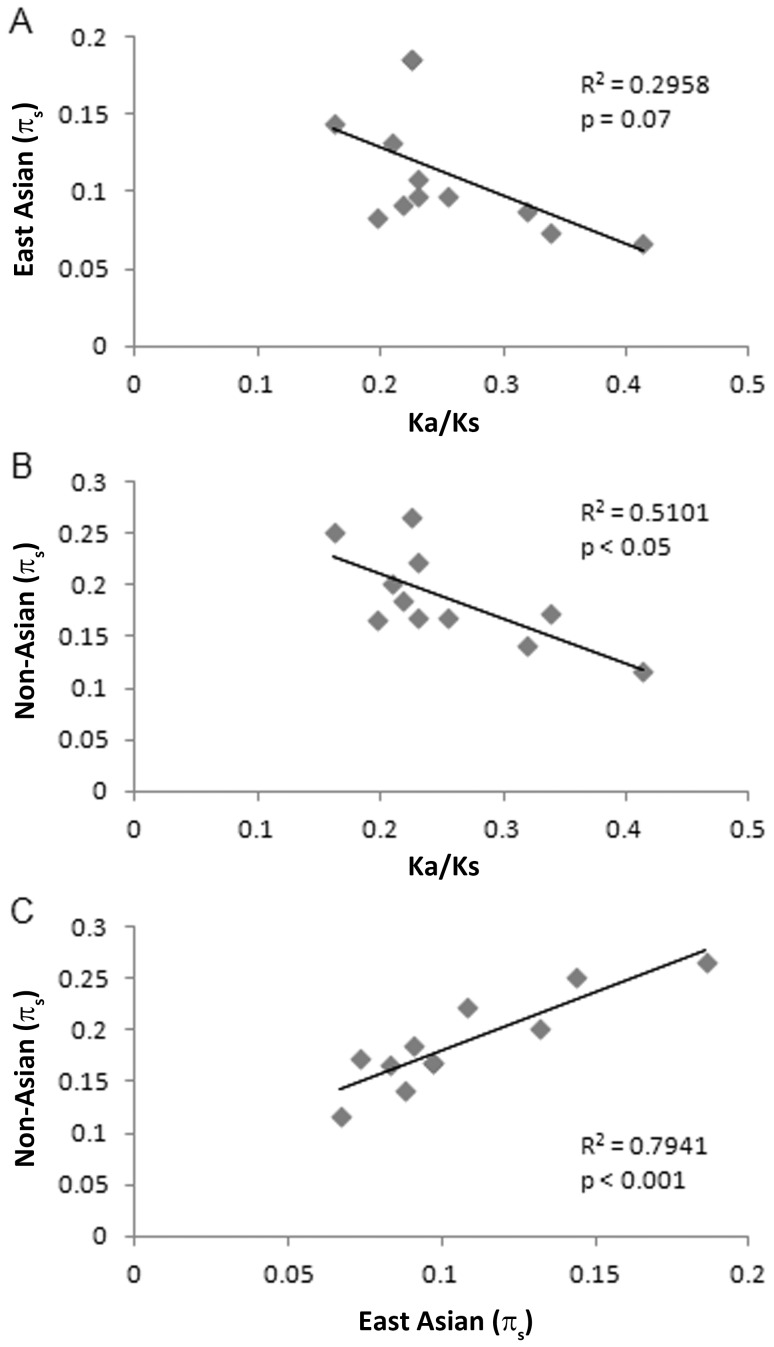
Correlation between Ka/Ks values and π_s_ values among 12 genes under diversifying selection, based on MKT analysis. (A,B) A linear regression analysis showed a significant correlation between Ka/Ks values and π_s_ values, when analyzing sequences from non-Asian strains (p<0.05). There was a non-significant correlation when analyzing these sequences from East Asian strains (p = 0.07). (C) There was a strong positive correlation when comparing π_s_ values of these 12 genes from either East Asian strains with the corresponding π_s_ values from non-Asian strains (p<0.001).

Among the divergent genes that were shown to be subject to positive diversifying selection based on the MKT, there was an inverse correlation when comparing π_s_ values to Ka/Ks values, and this was observed for both non-Asian and East Asian strains (Fig. 4). Thus, the genes with elevated Ka/Ks values have reduced silent site diversity due to positive selection. When comparing π_s_ values of the divergent genes isolated from non-Asian strains with the π_s_ values from the corresponding East Asian sequences, there was a significant positive correlation (p<0.001, Fig. 4), which indicates that the same genes experience reduced silent site diversity and increased positive selection in both East Asian and non-Asian populations. These results provide evidence that diversifying selection has spurred the rapid evolution of amino acid sequence changes in these two populations.

**Figure 5:**
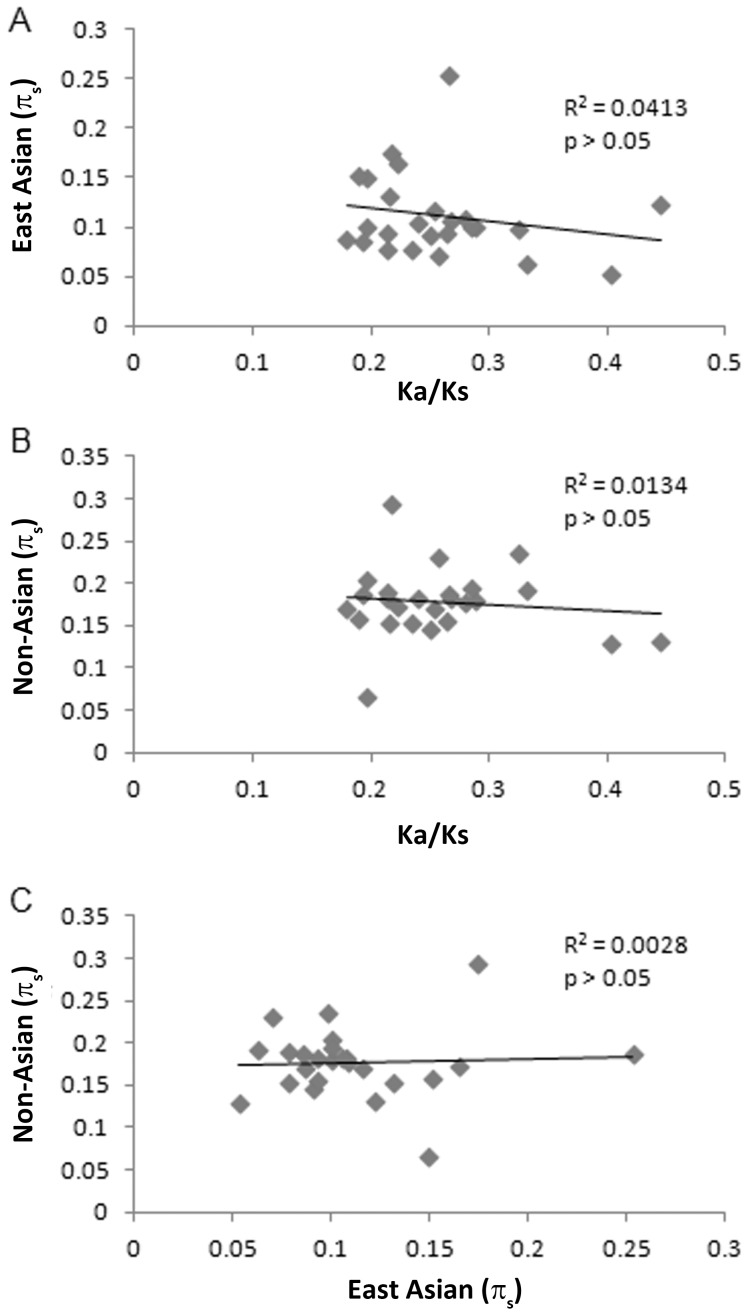
Lack of correlation between Ka/Ks values and π_s_ values among 25 genes that were not under diversifying selection, based on MKT analysis. (A,B) Linear regression analyses showed non-significant trends when comparing Ka/Ks values to π_s_ values (p>0.05). (C) There was no significant correlation between the π_s_ values of these sequences from East Asian strains with the corresponding π_s_ values from non-Asian strains (p>0.05).

We performed similar analyses for the set of genes that were not found to be under diversifying selection, based on MKT analysis (Fig. 5). In contrast to what was observed with the group of genes under diversifying selection (Fig. 4a and 4b), we did not detect any significant correlation between Ka/Ks values and π_s_ values when analyzing this group of genes. Thus, within this group of genes, as Ka/Ks ratios increase, the average π_s_ values across the gene do not change, which indicates that the genes are subject to more neutral evolution, as expected for the gene set that is not experiencing positive diversifying selection.

**Figure 6:**
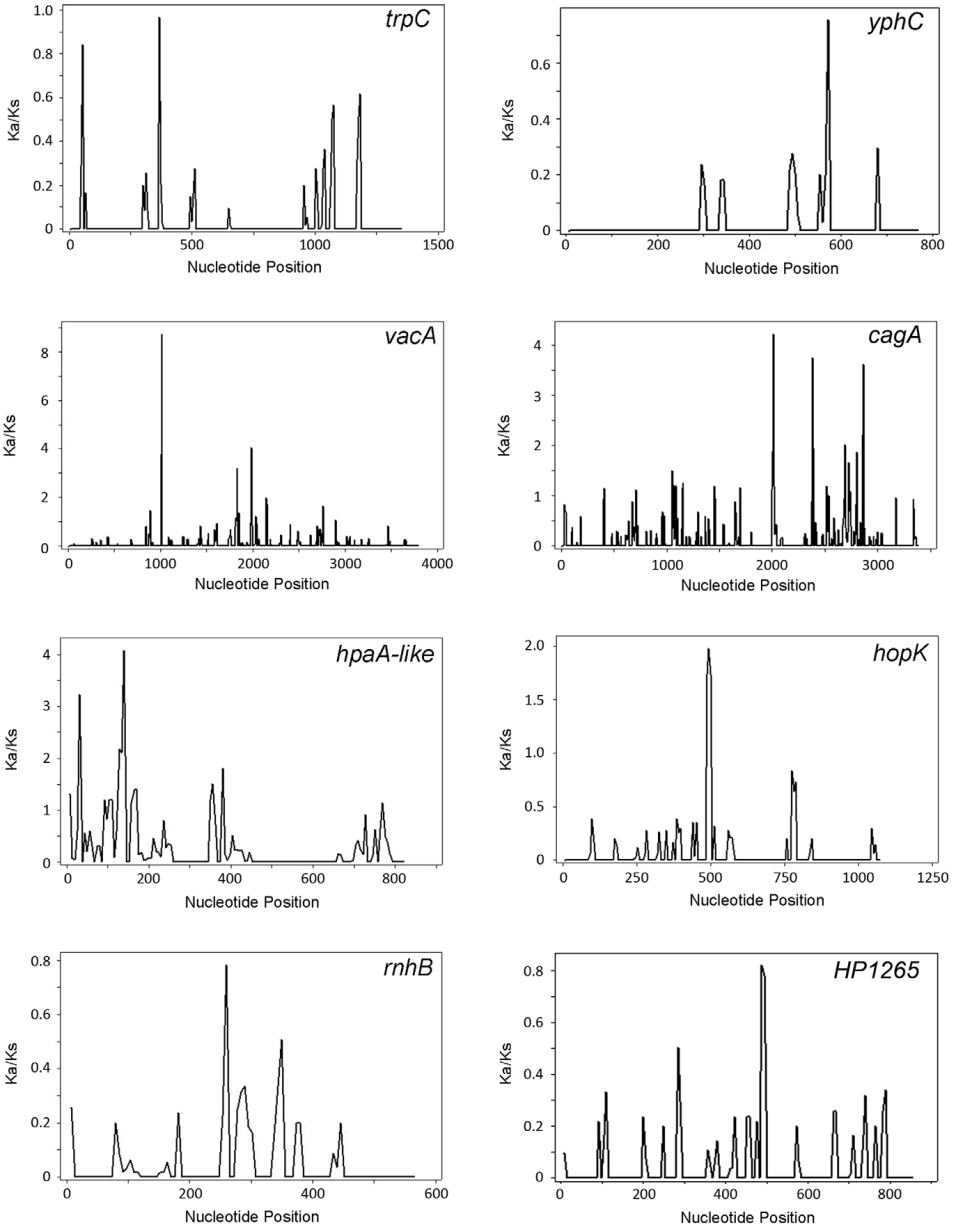
Sliding window analysis of positive selection (Ka/Ks) within selected genes. Sliding window analysis was performed to analyze the sequences of representative housekeeping genes (*trpC and yphC*) and several representative highly divergent genes, including *vacA* and *cagA* (under positive selection by MKT analysis) and an *hpaA-*like gene, *hopK, rnhB,* and HP1265 (not under positive selection by MKT analysis). Sequences from strain F16 (East Asian) and 26695 (non-Asian) were aligned and Ka/Ks ratios were calculated using DnaSP. In cases where sequences were not available from strains F16 or 26695, other representative East Asian or non-Asian sequences were analyzed. Parameters for the sliding window analysis were set at 50 bases (window size) and a step size of 10 bases. A Ka/Ks value of >1 indicates positive selection.

Gene-wide ratios of Ka/Ks are overly conservative because they do not reveal instances of positive selection at a few, specific amino acid sites in a protein sequence. Therefore, we next used site-by-site methods of detecting positive selection to analyze the group of 25 divergent genes that were not considered to be under diversifying selection based on MKT analysis. Overall, 20 out of the 25 genes exhibited at least one site with a Ka/Ks ratio above 1.0. Figure 6 shows a subset of the sliding window analyses, and illustrates that *vacA*, *cagA*, *hpaA-*like gene, and *hopK* have specific regions under positive selection, whereas *trpC* and *yphC* (housekeeping genes), *rnhB* and HP1265 do not.

## Discussion

*H. pylori* strains isolated in different parts of the world can be classified into distinct groups based on MLST analysis [13], [14]. Thus far, there has been relatively little comparative analysis of these groups at a whole-genome level. In this study, we undertook a systematic analysis designed to identify gene products that were highly divergent in East Asian *H. pylori* strains compared to non-Asian strains. Our analysis did not include a survey of gene deletions or gene disruptions in this panel of strains, but instead, we focused the analysis on genes that exhibited a high level of sequence divergence. We identified 57 predicted proteins, including CagA and VacA, with sequences that are highly divergent in East Asian *H. pylori* strains compared to non-Asian strains, and relatively conserved among the East Asian strains (Table 2). The most highly represented groups of divergent proteins were hypothetical proteins, cell envelope proteins, and proteins involved in DNA metabolism (Table 2). Among the 15 proteins classified in the cell envelope category, nine have been annotated as outer membrane proteins (OMPs).

Diversification of cell envelope proteins is of particular interest because of the important roles of the cell envelope in bacteria-host interactions. Three of the divergent outer membrane proteins identified in this study (HopZ, BabA and SabA) are reported to function as adhesins [54], [55], [56], and two other outer membrane proteins (HopA and HopD) are reported to have porin-like properties [57]. Two “VacA-like proteins” (HP0609/0610 and HP0922) were found to be highly divergent in East Asian strains compared to non-Asian strains. Similar to the vacuolating toxin VacA, these VacA-like proteins are predicted to be secreted by an autotransporter mechanism and localized to the bacteria surface [40]. The functions of these two VacA-like proteins and multiple other proteins annotated as “outer membrane proteins” have not yet been investigated. An HpaA-like protein (HP0492) was one of the most highly divergent proteins identified in this study. This protein of unknown function is considered a paralogue of HpaA, which in various studies has been reported to be either an adhesin or a flagellar-associated lipoprotein [58], [59]. Other divergent proteins within the cell envelope class [alpha-(1,3)-fucosyltransferase (FutB) and LPS 1,2-glucosyltransferase (RfaJ)] are predicted to have roles in LPS biosynthesis, and the former protein is reported to function as a molecular ruler for Lewis antigen biosynthesis [60]. Among the 15 divergent proteins in the cell envelope category, several (including RfaJ and the VacA-like proteins) are reported to be essential for *H. pylori* colonization of the stomach in animal models [61].

In addition to cell envelope proteins, other divergent proteins identified in this study are known or predicted to have important roles in bacteria-host interactions. For example, a protein originally annotated as “polyE-rich protein (HP0322)” and renamed “ChePep” was recently shown to be critical for *H. pylori* chemotaxis and is required for *H. pylori* colonization of deep gastric glands [62]. One of the proteins originally classified as a hypothetical protein (HP0721) is a secreted protein that has sialic acid-binding properties [63].

Numerous highly divergent proteins identified in this study are predicted to have functions related to DNA metabolism (including DNA replication, recombination and repair and restriction-modification). *H. pylori* is recombinogenic and is naturally competent for the uptake of DNA [64]. The ability to undergo DNA uptake and recombination promotes diversification of *H. pylori* and may allow the bacteria to adapt rapidly to the gastric environment of new hosts or changing conditions within a host. Several genes required for DNA uptake and recombination have important roles in promoting *H. pylori* colonization of the stomach in animal models [65], [66]. In addition, restriction-modification genes presumably have an important role in protecting *H. pylori* against phage and plasmids, and may promote the preferential uptake and chromosomal integration of *H. pylori* DNA rather than exogenous DNA from other sources. Interestingly, comparative genomic analyses of *Neisseria* isolates have also revealed marked differences among strains in restriction modification systems, and it was proposed that these systems may have a role in limiting gene flow [67].

Several previous studies have detected genes or proteins that are divergent in East Asian strains compared to non-Asian strains. For example, in a previous study, we analyzed the genome of a single East Asian strain (98-10) and detected 8 encoded proteins that were highly divergent compared to orthologues encoded by 3 non-Asian genomes that were available at the time [35]. A recent study analyzed multiple East Asian strains and reported the identification of additional divergent genes [37]. The use of different methodology in the current study allowed us to identify a set of divergent genes that partially overlaps those identified in these previous studies, and also includes multiple divergent genes that have not been previously recognized.

In previous studies, several proteins within the Sel1-like (SLR) gene family were reported to be highly divergent when comparing gene sequences from *H. pylori* strains classified as African, East Asian, and European, and it was reported that positive selection has driven the divergence of these proteins [36]. Most of these Sel1-like proteins were not identified in the current analysis due to the stringent criteria that we used for detecting proteins that were highly divergent in East Asian and non-Asian populations and for detecting proteins that were conserved within the East Asian population (described in Methods). For example, HP0519 is highly divergent in *H. pylori* strains from Japan compared to non-Asian *H. pylori* strains, but orthologous sequences in Korean strains resemble those of non-Asian strains [36]; therefore, this protein did not meet the criteria utilized in the current study.

Previous studies have shown that the divergence of two important virulence determinants of *H. pylori*, *cagA* and *vacA*, has been driven by positive selection [18], [23], [25], [68]. Similarly, we show, based on an analysis of Ka/Ks ratios, that most of the divergent genes with known or predicted functions exhibit evidence of positive selection. Furthermore, we show, based on MKT analysis, that 12 of the divergent genes, including *cagA* and *vacA*, are under positive, diversifying selection. Consistent with what one would expect, we show that genes subject to diversifying selection often exhibit a reduction in effective population size, as estimated by π_s_ analysis. Among the 37 divergent genes with known or predicted functions, 32 (86%) exhibit evidence of positive selection, based on the MKT analysis combined with the sliding window analyses of Ka/Ks.

As illustrated in Figure 1, there was a higher level of intragroup relatedness among East Asian strains than among non-East Asian strains. Correspondingly, the mean silent site diversities in the divergent gene set were 0.11 and 0.18 for East Asian and non-East Asian strains, respectively, and in the control housekeeping set were 0.08 and 0.15 for East Asian and non-East Asian strains, respectively (Table 3). There was also a trend toward higher levels of silent site diversity in the divergent genes than in the control housekeeping genes. Specifically, the average silent site diversities were 0.15 and 0.11 for the divergent and control gene sets, respectively. There are at least three possible explanations for why there is an average increased rate of silent site diversity in the divergent gene set. One possibility is that there might be differences in recombination within control and divergent gene sets. To address this possibility, we tested for the presence of recombination within each nucleotide alignment using the program PHItest. An evaluation of the performance of several recombination programs using both simulated and empirical data found that PHItest effectively determines recombination under diverse conditions and performs markedly better than Max v2 and NSS at avoiding false positives of recombination under models of substitution rate heterogeneity [69]. The results of this analysis showed that all six of the divergent genes analyzed in Figure 2 and six out of seven genes in the control gene set exhibit significant recombination. Thus, recombination is widespread in *H. pylori*, as expected, and wholesale differences in recombination between the divergent and control gene sets do not explain the average increase in silent site diversity in the divergent gene set. An alternative explanation is that slight differences in genetic drift could explain the increase in silent site diversity in the divergent gene set. For example, the control gene set is composed of housekeeping genes under constant levels of purifying/negative selection. Neutral substitutions can accumulate under this form of selection but silent sites linked to negatively selected amino acid changes will be purified. In contrast, the divergent set experienced bouts of positive selection that were intermittent over the course of the gene’s evolution and localized to certain amino acid positions rather than the entire gene. Thus, bouts of adaptive evolution could be followed by bouts of neutral evolution across different gene regions, leading to the observed average increase in silent site diversity. Finally, a third possible explanation for why there is a slight increase in silent site diversity in the divergent genes is that mutation rates can differ between different genes within the same genome [70].

We hypothesize that in many cases, genes which are highly divergent in East Asian strains compared to non-Asian strains encode proteins that differ in functional activity. Several examples of this phenomenon have been reported previously. For example, East Asian forms of CagA (containing EPIYA-D motifs) are reported to differ in activity compared to Western forms of CagA (containing EPIYA-C motifs) [31]. Similarly, East Asian forms of SabA are reported to differ in activity compared to non-Asian forms of SabA [34]. We speculate that East Asian *H. pylori* strains are subject to different host or environmental conditions compared to non-Asian strains, and these conditions may have driven diversification of certain genes. Consistent with this hypothesis, diversification of OMPs has been associated with the adaptation of *H. pylori* to different host selective pressures [71], [72].

Finally, it is notable that the incidence of gastric cancer is markedly higher in many parts of East Asia than in non-Asian countries [5], [7], [8]. Distinctive properties of *H. pylori* strains circulating in various regions of East Asia may contribute to this high incidence of gastric cancer. As one example, forms of CagA containing an EPIYA-D motif (which are found in East Asian strains) exhibit increased activity *in vitro* compared to other forms of CagA [30], [31]. It seems possible that other proteins encoded by East Asian strains may differ in activity compared to the corresponding proteins encoded by non-East Asian strains, and such differences in activity may influence pathologic processes linked to the development of gastric cancer. In future studies, it will be important to specifically test the functional roles of the divergent proteins identified in this study, to investigate their role in *H. pylori*-host interactions, and to investigate their potential roles as determinants of gastric cancer risk. In addition, it will be important to investigate further the diversity of these genes in other geographic populations of *H. pylori* strains.

## Supporting Information

Figure S1 Phylogenetic analysis of *cagA, vacA,* and housekeeping gene sequences. Neighbor-joining phylogenetic trees were constructed for *cagA* (panel A) and *vacA* (panel B), and a set of seven concatenated housekeeping gene fragments (panel C). The sequences of East Asian strains (boxed) are highly divergent when compared to corresponding nucleotide sequences of non-Asian strains. (Note the difference in scales used for the three trees).
(TIF)

Table S1 Analysis of nucleotide diversity (outliers removed).
(DOCX)

Table S2 Analysis of positive selection using McDonald-Kreitman test (outliers removed).
(DOCX)

## References

[1] Atherton JC, Blaser MJ (2009) Coadaptation of *Helicobacter pylori* and humans: ancient history, modern implications. J Clin Invest 119: 2475–2487. 19729845 10.1172/JCI38605 PMC2735910

[2] Cover TL, Blaser MJ (2009) *Helicobacter pylori* in health and disease. Gastroenterology 136: 1863–1873. 19457415 10.1053/j.gastro.2009.01.073 PMC3644425

[3] Amieva MR, El-Omar EM (2008) Host-bacterial interactions in *Helicobacter pylori* infection. Gastroenterology 134: 306–323. 18166359 10.1053/j.gastro.2007.11.009

[4] Kusters JG, van Vliet AH, Kuipers EJ (2006) Pathogenesis of *Helicobacter pylori* infection. Clin Microbiol Rev 19: 449–490. 16847081 10.1128/CMR.00054-05 PMC1539101

[5] Fuchs CS, Mayer RJ (1995) Gastric carcinoma. N Engl J Med 333: 32–41. 7776992 10.1056/NEJM199507063330107

[6] de Martel C, Ferlay J, Franceschi S, Vignat J, Bray F, et al (2012) Global burden of cancers attributable to infections in 2008: a review and synthetic analysis. Lancet Oncol 13: 607–615. 22575588 10.1016/S1470-2045(12)70137-7

[7] Leung WK, Wu MS, Kakugawa Y, Kim JJ, Yeoh KG, et al (2008) Screening for gastric cancer in Asia: current evidence and practice. Lancet Oncol 9: 279–287. 18308253 10.1016/S1470-2045(08)70072-X

[8] Uemura N, Okamoto S, Yamamoto S, Matsumura N, Yamaguchi S, et al (2001) *Helicobacter pylori* infection and the development of gastric cancer. N Engl J Med 345: 784–789. 11556297 10.1056/NEJMoa001999

[9] Herrera V, Parsonnet J (2009) *Helicobacter pylori* and gastric adenocarcinoma. Clin Microbiol Infect 15: 971–976. 19874380 10.1111/j.1469-0691.2009.03031.x

[10] Blaser MJ, Berg DE (2001) *Helicobacter pylori* genetic diversity and risk of human disease. J Clin Invest 107: 767–773. 11285290 10.1172/JCI12672 PMC199587

[11] Suerbaum S, Josenhans C (2007) *Helicobacter pylori* evolution and phenotypic diversification in a changing host. Nat Rev Microbiol 5: 441–452. 17505524 10.1038/nrmicro1658

[12] Suzuki R, Shiota S, Yamaoka Y (2012) Molecular epidemiology, population genetics, and pathogenic role of *Helicobacter pylori* . Infect Genet Evol 12: 203–213. 22197766 10.1016/j.meegid.2011.12.002 PMC3294018

[13] Falush D, Wirth T, Linz B, Pritchard JK, Stephens M, et al (2003) Traces of human migrations in *Helicobacter pylori* populations. Science 299: 1582–1585. 12624269 10.1126/science.1080857

[14] Moodley Y, Linz B, Yamaoka Y, Windsor HM, Breurec S, et al (2009) The peopling of the Pacific from a bacterial perspective. Science 323: 527–530. 19164753 10.1126/science.1166083 PMC2827536

[15] Kersulyte D, Kalia A, Gilman RH, Mendez M, Herrera P, et al (2010) *Helicobacter pylori* from Peruvian amerindians: traces of human migrations in strains from remote Amazon, and genome sequence of an Amerind strain. PLoS ONE 5: e15076. 21124785 10.1371/journal.pone.0015076 PMC2993954

[16] Linz B, Balloux F, Moodley Y, Manica A, Liu H, et al (2007) An African origin for the intimate association between humans and *Helicobacter pylori* . Nature 445: 915–918. 17287725 10.1038/nature05562 PMC1847463

[17] Moodley Y, Linz B, Bond RP, Nieuwoudt M, Soodyall H, et al (2012) Age of the association between *Helicobacter pylori* and man. PLoS Pathog 8: e1002693. 22589724 10.1371/journal.ppat.1002693 PMC3349757

[18] Gangwer KA, Shaffer CL, Suerbaum S, Lacy DB, Cover TL, et al (2010) Molecular evolution of the *Helicobacter pylori* vacuolating toxin gene *vacA* . J Bacteriol 192: 6126–6135. 20870762 10.1128/JB.01081-10 PMC2981223

[19] Cover TL, Blanke SR (2005) *Helicobacter pylori* VacA, a paradigm for toxin multifunctionality. Nat Rev Microbiol 3: 320–332. 15759043 10.1038/nrmicro1095

[20] Hatakeyama M (2004) Oncogenic mechanisms of the *Helicobacter pylori* CagA protein. Nat Rev Cancer 4: 688–694. 15343275 10.1038/nrc1433

[21] Hatakeyama M (2011) Anthropological and clinical implications for the structural diversity of the *Helicobacter pylori* CagA oncoprotein. Cancer Sci 102: 36–43. 20942897 10.1111/j.1349-7006.2010.01743.x PMC11159401

[22] Ohnishi N, Yuasa H, Tanaka S, Sawa H, Miura M, et al. (2008) Transgenic expression of *Helicobacter pylori* CagA induces gastrointestinal and hematopoietic neoplasms in mouse. Proc Natl Acad Sci U S A. 10.1073/pnas.0711183105 PMC2242726 18192401

[23] Olbermann P, Josenhans C, Moodley Y, Uhr M, Stamer C, et al (2010) A global overview of the genetic and functional diversity in the *Helicobacter pylori* cag pathogenicity island. PLoS Genet 6: e1001069. 20808891 10.1371/journal.pgen.1001069 PMC2924317

[24] Fischer W (2011) Assembly and molecular mode of action of the *Helicobacter pylori* Cag type IV secretion apparatus. FEBS J 278: 1203–1212. 21352490 10.1111/j.1742-4658.2011.08036.x

[25] Duncan SS, Valk PL, Shaffer CL, Bordenstein SR, Cover TL (2012) J-Western forms of *Helicobacter pylori cagA* constitute a distinct phylogenetic group with a widespread geographic distribution. J Bacteriol 194: 1593–1604. 22247512 10.1128/JB.06340-11 PMC3294863

[26] Atherton JC, Cao P, Peek RM Jr, Tummuru MK, Blaser MJ, et al (1995) Mosaicism in vacuolating cytotoxin alleles of *Helicobacter pylori*. Association of specific *vacA* types with cytotoxin production and peptic ulceration. J Biol Chem 270: 17771–17777. 7629077 10.1074/jbc.270.30.17771

[27] Blaser MJ, Perez-Perez GI, Kleanthous H, Cover TL, Peek RM, et al (1995) Infection with *Helicobacter pylori* strains possessing *cagA* is associated with an increased risk of developing adenocarcinoma of the stomach. Cancer Res 55: 2111–2115. 7743510

[28] Figueiredo C, Machado JC, Pharoah P, Seruca R, Sousa S, et al (2002) *Helicobacter pylori* and interleukin 1 genotyping: an opportunity to identify high-risk individuals for gastric carcinoma. J Natl Cancer Inst 94: 1680–1687. 12441323 10.1093/jnci/94.22.1680

[29] Ito Y, Azuma T, Ito S, Miyaji H, Hirai M, et al (1997) Analysis and typing of the *vacA* gene from *cagA*-positive strains of *Helicobacter pylori* isolated in Japan. J Clin Microbiol 35: 1710–1714. 9196179 10.1128/jcm.35.7.1710-1714.1997 PMC229827

[30] Higashi H, Tsutsumi R, Fujita A, Yamazaki S, Asaka M, et al (2002) Biological activity of the *Helicobacter pylori* virulence factor CagA is determined by variation in the tyrosine phosphorylation sites. Proc Natl Acad Sci U S A 99: 14428–14433. 12391297 10.1073/pnas.222375399 PMC137900

[31] Naito M, Yamazaki T, Tsutsumi R, Higashi H, Onoe K, et al (2006) Influence of EPIYA-repeat polymorphism on the phosphorylation-dependent biological activity of *Helicobacter pylori* CagA. Gastroenterology 130: 1181–1190. 16618412 10.1053/j.gastro.2005.12.038

[32] Van Doorn LJ, Figueiredo C, Megraud F, Pena S, Midolo P, et al (1999) Geographic distribution of *vacA* allelic types of *Helicobacter pylori* . Gastroenterology 116: 823–830. 10092304 10.1016/s0016-5085(99)70065-x

[33] van Doorn LJ, Figueiredo C, Sanna R, Pena S, Midolo P, et al (1998) Expanding allelic diversity of *Helicobacter pylori vacA* . J Clin Microbiol 36: 2597–2603. 9705399 10.1128/jcm.36.9.2597-2603.1998 PMC105169

[34] Lu H, Wu JY, Beswick EJ, Ohno T, Odenbreit S, et al (2007) Functional and intracellular signaling differences associated with the *Helicobacter pylori* AlpAB adhesin from Western and East Asian strains. J Biol Chem 282: 6242–6254. 17202133 10.1074/jbc.M611178200 PMC3130062

[35] McClain MS, Shaffer CL, Israel DA, Peek RM Jr, Cover TL (2009) Genome sequence analysis of *Helicobacter pylori* strains associated with gastric ulceration and gastric cancer. BMC Genomics 10: 3. 19123947 10.1186/1471-2164-10-3 PMC2627912

[36] Ogura M, Perez JC, Mittl PR, Lee HK, Dailide G, et al (2007) *Helicobacter pylori* evolution: lineage- specific adaptations in homologs of eukaryotic Sel1-like genes. PLoS Comput Biol 3: e151. 17696605 10.1371/journal.pcbi.0030151 PMC1941758

[37] Kawai M, Furuta Y, Yahara K, Tsuru T, Oshima K, et al (2011) Evolution in an oncogenic bacterial species with extreme genome plasticity: *Helicobacter pylori* East Asian genomes. BMC Microbiol 11: 104. 21575176 10.1186/1471-2180-11-104 PMC3120642

[38] Tamura K, Dudley J, Nei M, Kumar S (2007) MEGA4: Molecular Evolutionary Genetics Analysis (MEGA) software version 4.0. Mol Biol Evol 24: 1596–1599. 17488738 10.1093/molbev/msm092

[39] Furuta Y, Kawai M, Yahara K, Takahashi N, Handa N, et al (2011) Birth and death of genes linked to chromosomal inversion. Proc Natl Acad Sci U S A 108: 1501–1506. 21212362 10.1073/pnas.1012579108 PMC3029772

[40] Tomb J-F, White O, Kerlavage AR, Clayton RA, Sutton GG, et al (1997) The complete genome sequence of the gastric pathogen *Helicobacter pylori* . Nature 388: 539–547. 9252185 10.1038/41483

[41] Alm RA, Ling LS, Moir DT, King BL, Brown ED, et al (1999) Genomic-sequence comparison of two unrelated isolates of the human gastric pathogen *Helicobacter pylori* . Nature 397: 176–180. 9923682 10.1038/16495

[42] Oh JD, Kling-Backhed H, Giannakis M, Xu J, Fulton RS, et al (2006) The complete genome sequence of a chronic atrophic gastritis *Helicobacter pylori* strain: evolution during disease progression. Proc Natl Acad Sci U S A 103: 9999–10004. 16788065 10.1073/pnas.0603784103 PMC1480403

[43] Baltrus DA, Amieva MR, Covacci A, Lowe TM, Merrell DS, et al (2009) The complete genome sequence of *Helicobacter pylori* strain G27. J Bacteriol 191: 447–448. 18952803 10.1128/JB.01416-08 PMC2612421

[44] Fischer W, Windhager L, Rohrer S, Zeiller M, Karnholz A, et al (2010) Strain-specific genes of *Helicobacter pylori*: genome evolution driven by a novel type IV secretion system and genomic island transfer. Nucleic Acids Res 38: 6089–6101. 20478826 10.1093/nar/gkq378 PMC2952849

[45] Farnbacher M, Jahns T, Willrodt D, Daniel R, Haas R, et al (2010) Sequencing, annotation, and comparative genome analysis of the gerbil-adapted *Helicobacter pylori* strain B8. BMC Genomics 11: 335. 20507619 10.1186/1471-2164-11-335 PMC3091624

[46] Thiberge JM, Boursaux-Eude C, Lehours P, Dillies MA, Creno S, et al (2010) From array-based hybridization of *Helicobacter pylori* isolates to the complete genome sequence of an isolate associated with MALT lymphoma. BMC Genomics 11: 368. 20537153 10.1186/1471-2164-11-368 PMC3091627

[47] Devi SH, Taylor TD, Avasthi TS, Kondo S, Suzuki Y, et al (2010) Genome of *Helicobacter pylori* strain 908. J Bacteriol 192: 6488–6489. 20952566 10.1128/JB.01110-10 PMC3008520

[48] Stajich JE, Block D, Boulez K, Brenner SE, Chervitz SA, et al (2002) The Bioperl toolkit: Perl modules for the life sciences. Genome Res 12: 1611–1618. 12368254 10.1101/gr.361602 PMC187536

[49] Rasko DA, Myers GS, Ravel J (2005) Visualization of comparative genomic analyses by BLAST score ratio. BMC Bioinformatics 6: 2. 15634352 10.1186/1471-2105-6-2 PMC545078

[50] Yao J, Lin H, Doddapaneni H, Civerolo EL (2007) nWayComp: a genome-wide sequence comparison tool for multiple strains/species of phylogenetically related microorganisms. In Silico Biol 7: 195–200. 17688445

[51] Abascal F, Zardoya R, Posada D (2005) ProtTest: selection of best-fit models of protein evolution. Bioinformatics 21: 2104–2105. 15647292 10.1093/bioinformatics/bti263

[52] Ronquist F, Teslenko M, van der Mark P, Ayres DL, Darling A, et al (2012) MrBayes 3.2: efficient Bayesian phylogenetic inference and model choice across a large model space. Syst Biol 61: 539–542. 22357727 10.1093/sysbio/sys029 PMC3329765

[53] McDonald JH, Kreitman M (1991) Adaptive protein evolution at the Adh locus in Drosophila. Nature 351: 652–654. 1904993 10.1038/351652a0

[54] Ilver D, Arnqvist A, Ogren J, Frick IM, Kersulyte D, et al (1998) *Helicobacter pylori* adhesin binding fucosylated histo-blood group antigens revealed by retagging. Science 279: 373–377. 9430586 10.1126/science.279.5349.373

[55] Mahdavi J, Sonden B, Hurtig M, Olfat FO, Forsberg L, et al (2002) *Helicobacter pylori* SabA adhesin in persistent infection and chronic inflammation. Science 297: 573–578. 12142529 10.1126/science.1069076 PMC2570540

[56] Peck B, Ortkamp M, Diehl KD, Hundt E, Knapp B (1999) Conservation, localization and expression of HopZ, a protein involved in adhesion of *Helicobacter pylori* . Nucleic Acids Res 27: 3325–3333. 10454640 10.1093/nar/27.16.3325 PMC148566

[57] Exner MM, Doig P, Trust TJ, Hancock RE (1995) Isolation and characterization of a family of porin proteins from *Helicobacter pylori* . Infect Immun 63: 1567–1572. 7534278 10.1128/iai.63.4.1567-1572.1995 PMC173190

[58] O’Toole PW, Janzon L, Doig P, Huang J, Kostrzynska M, et al (1995) The putative neuraminyllactose-binding hemagglutinin HpaA of *Helicobacter pylori* CCUG 17874 is a lipoprotein. J Bacteriol 177: 6049–6057. 7592366 10.1128/jb.177.21.6049-6057.1995 PMC177441

[59] Jones AC, Logan RP, Foynes S, Cockayne A, Wren BW, et al (1997) A flagellar sheath protein of *Helicobacter pylori* is identical to HpaA, a putative N-acetylneuraminyllactose-binding hemagglutinin, but is not an adhesin for AGS cells. J Bacteriol 179: 5643–5647. 9287032 10.1128/jb.179.17.5643-5647.1997 PMC179448

[60] Nilsson C, Skoglund A, Moran AP, Annuk H, Engstrand L, et al (2006) An enzymatic ruler modulates Lewis antigen glycosylation of *Helicobacter pylori* LPS during persistent infection. Proc Natl Acad Sci U S A 103: 2863–2868. 16477004 10.1073/pnas.0511119103 PMC1413829

[61] Kavermann H, Burns BP, Angermuller K, Odenbreit S, Fischer W, et al (2003) Identification and characterization of *Helicobacter pylori* genes essential for gastric colonization. J Exp Med 197: 813–822. 12668646 10.1084/jem.20021531 PMC2193887

[62] Howitt MR, Lee JY, Lertsethtakarn P, Vogelmann R, Joubert LM, et al. (2011) ChePep controls *Helicobacter pylori* infection of the gastric glands and chemotaxis in the Epsilonproteobacteria. MBio 2. 10.1128/mBio.00098-11 PMC3143842 21791582

[63] Bennett HJ, Roberts IS (2005) Identification of a new sialic acid-binding protein in *Helicobacter pylori* . FEMS Immunol Med Microbiol 44: 163–169. 15866211 10.1016/j.femsim.2004.11.008

[64] Suerbaum S, Smith JM, Bapumia K, Morelli G, Smith NH, et al (1998) Free recombination within *Helicobacter pylori* . Proc Natl Acad Sci U S A 95: 12619–12624. 9770535 10.1073/pnas.95.21.12619 PMC22880

[65] Amundsen SK, Fero J, Hansen LM, Cromie GA, Solnick JV, et al (2008) *Helicobacter pylori* AddAB helicase-nuclease and RecA promote recombination-related DNA repair and survival during stomach colonization. Mol Microbiol 69: 994–1007. 18573180 10.1111/j.1365-2958.2008.06336.x PMC2680919

[66] Wang G, Maier RJ (2009) A RecB-like helicase in *Helicobacter pylori* is important for DNA repair and host colonization. Infect Immun 77: 286–291. 18981252 10.1128/IAI.00970-08 PMC2612261

[67] Maiden MC (2008) Population genomics: diversity and virulence in the Neisseria. Curr Opin Microbiol 11: 467–471. 18822386 10.1016/j.mib.2008.09.002 PMC2612085

[68] Torres-Morquecho A, Giono-Cerezo S, Camorlinga-Ponce M, Vargas-Mendoza CF, Torres J (2010) Evolution of bacterial genes: evidences of positive Darwinian selection and fixation of base substitutions in virulence genes of *Helicobacter pylori* . Infect Genet Evol 10: 764–776. 20434592 10.1016/j.meegid.2010.04.005

[69] Bruen TC, Philippe H, Bryant D (2006) A simple and robust statistical test for detecting the presence of recombination. Genetics 172: 2665–2681. 16489234 10.1534/genetics.105.048975 PMC1456386

[70] Martincorena I, Seshasayee AS, Luscombe NM (2012) Evidence of non-random mutation rates suggests an evolutionary risk management strategy. Nature 485: 95–98. 22522932 10.1038/nature10995

[71] Aspholm-Hurtig M, Dailide G, Lahmann M, Kalia A, Ilver D, et al (2004) Functional adaptation of BabA, the *Helicobacter pylori* ABO blood group antigen binding adhesin. Science 305: 519–522. 15273394 10.1126/science.1098801

[72] Kennemann L, Didelot X, Aebischer T, Kuhn S, Drescher B, et al (2011) *Helicobacter pylori* genome evolution during human infection. Proc Natl Acad Sci U S A 108: 5033–5038. 21383187 10.1073/pnas.1018444108 PMC3064335

