# Supplementary figures and images for: Comparative Genomic Analysis of East Asian and Non-Asian Helicobacter pylori Strains Identifies Rapidly Evolving Genes
Source: PLoS One. 2013 Jan 31;8(1):e55120. doi: 10.1371/journal.pone.0055120 (PMC3561388; doi:10.1371/journal.pone.0055120)

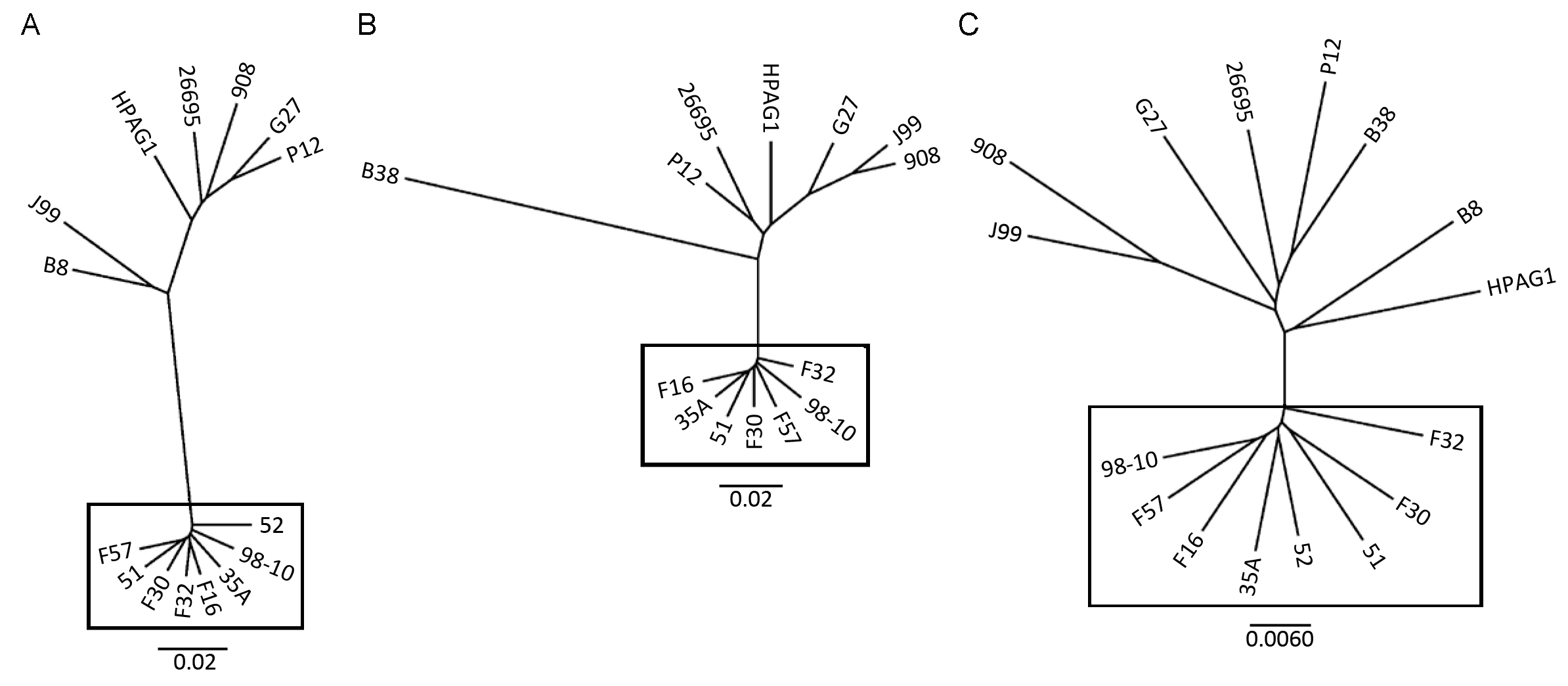

Supplement: Figure S1 — Phylogenetic analysis of cagA, vacA, and housekeeping gene sequences. Neighbor-joining phylogenetic trees were constructed for cagA (panel A) and vacA (panel B), and a set of seven concatenated housekeeping gene fragments (panel C). The sequences of East Asian strains (boxed) are highly divergent when compared to corresponding nucleotide sequences of non-Asian strains. (Note the difference in scales used for the three trees). (TIF) [file pone.0055120.s001.tif]
