# Supplementary material for: Comparative Genomic Analysis of East Asian and Non-Asian Helicobacter pylori Strains Identifies Rapidly Evolving Genes
Source: PLoS One. 2013 Jan 31;8(1):e55120. doi: 10.1371/journal.pone.0055120 (PMC3561388; doi:10.1371/journal.pone.0055120)
Supplement: Table S1 — Analysis of nucleotide diversity (outliers removed). (DOCX) [file pone.0055120.s002.docx]

Table S1. Analysis of nucleotide diversity (outliers removed) ^a^

| **Main role** | **Annotation** | **Gene ID (26695)** | **Mean % aa identity (EA vs. Non-EA) ^b^** | **π_a_-EA** | **π_a_-Non EA** | **π_s_-EA** | **π_s_-Non EA** | **Ka/Ks (EA-NEA)^c^** |
| --- | --- | --- | --- | --- | --- | --- | --- | --- |
| Cell envelope | outer membrane protein HopZ (omp1) | HP0009 | 71.65 | 0.070 | 0.039 | 0.253 | 0.186 | 0.265 |
| Cell envelope | outer membrane protein HopD (omp2) | HP0025 | 87.92 | 0.015 | 0.019 | 0.097 | 0.169 | 0.254 |
| Cell envelope | outer membrane protein BabA (omp28) | HP1243 | 87.65 | - | - | - | - | - |
| Cell envelope | outer membrane protein HomC/HomD | HP0373 | 74.54 | 0.012 | 0.026 | 0.073 | 0.173 | 0.338 |
| Cell envelope | outer membrane protein HomB | NA^d^ | 87.06 | 0.016 | 0.038 | 0.108 | 0.222 | 0.230 |
| Cell envelope | outer membrane protein SabA/HopP/sialic acid-binding adhesin (omp17) | HP0725 | 87.12 | 0.036 | 0.039 | 0.107 | 0.183 | 0.267 |
| Cell envelope | outer membrane protein HopK (omp12) | HP0923 | 89.23 | - | - | - | - | **-** |
| Cell envelope | outer membrane protein HopA (omp6) | HP0229 | 89.69 | 0.041 | 0.044 | 0.116 | 0.171 | 0.253 |
| Cell envelope | outer membrane protein HopL (omp26) | HP1157 | 89.58 | - | - | - | - | **-** |
| Cell envelope | vacuolating cytotoxin (VacA)-like protein | HP0609/0610 | 91.09 | - | - | - | - | - |
| Cell envelope | vacuolating cytotoxin (VacA)-like protein | HP0922 | 89.93 | - | - | - | - | **-** |
| Cell envelope | hpaA-like protein | HP0492 | 71.03 | - | - | - | - | **-** |
| Cell envelope | alpha-(1,3)-fucosyltransferase | HP0651 | 81.68 | - | - | - | - | - |
| Cell envelope | lipopolysaccharide 1,2-glucosyltransferase (rfaJ) | HP0159 | 86.99 | - | - | - | - | - |
| Cell envelope | cysteine-rich protein D/beta-lactamase HcpD | HP0160 | 90.00 | 0.022 | 0.025 | 0.088 | 0.100 | 0.338 |
| Cellular processes | cytotoxin associated protein A (cagA) | HP0547 | 77.06 | 0.018 | 0.038 | 0.067 | 0.093 | 0.403 |
| Cellular processes | vacuolating cytotoxin A | HP0887 | 89.26 | 0.013 | 0.034 | 0.097 | 0.169 | 0.231 |
| Cellular processes | Flagellar hook-length control protein | HP0906 | 85.76 | - | - | - | - | - |
| DNA metabolism | recombination protein RecB/helicase | HP1553 | 89.52 | - | - | - | - | - |
| DNA metabolism | ribonuclease H (rnhA) | HP0661 | 82.59 | 0.020 | 0.019 | 0.149 | 0.066 | 0.197 |
| DNA metabolism | ribonuclease HII (rnhB) | HP1323 | 87.90 | - | - | - | - | **-** |
| DNA metabolism | type I restriction enzyme M protein/ HsdM | HP0463 | 89.23 | - | - | - | - | - |
| DNA metabolism | Type I restriction enzyme M protein (HsdM) | HP0850 | 89.79 | 0.026 | 0.027 | 0.131 | 0.154 | 0.215 |
| DNA metabolism | Type IIG restriction-modification enzyme/adenine specific DNA methyltransferase | HP1354 | 81.94 | 0.031 | 0.069 | 0.098 | 0.235 | 0.324 |
| DNA metabolism | type III restriction enzyme R protein | HP1371 | 83.71 | - | - | - | - | - |
| Protein fate | metalloprotease | HP0806 | 87.47 | 0.016 | 0.047 | 0.070 | 0.231 | 0.257 |
| Protein fate | preprotein translocase subunit SecG | HP1255 | 89.15 | - | - | - | - | - |
| Protein synthesis | tRNA delta(2)-isopentenylpyrophosphate transferase (miaA) | HP1415 | 79.88 | 0.020 | 0.049 | 0.100 | 0.179 | 0.288 |
| Protein synthesis | selenocysteine synthase (SelA)/L-seryl-tRNA(Sec) selenium transferase | HP1513 | 88.70 | 0.024 | 0.033 | 0.093 | 0.181 | 0.214 |
| Purines, pyrimidines, nucleosides, and nucleotides | purine nucleoside phosphorylase (punB) | HP1530 | 88.73 | 0.016 | 0.033 | 0.091 | 0.185 | 0.218 |
| Transcription | poly(A) polymerase (papS) | HP0640 | 88.85 | 0.018 | 0.034 | 0.085 | 0.187 | 0.193 |
| Unknown function | poly E-rich protein | HP0322 | 71.69 | 0.027 | 0.046 | 0.105 | 0.183 | 0.239 |
| Hypothetical proteins | tRNA(Ile)-lysidine synthase | HP0728 | 89.74 | 0.017 | 0.034 | 0.078 | 0.152 | 0.234 |
| Hypothetical proteins | probable ATP /GTP binding protein | HP0729 | 88.15 | - | - | - | - | - |
| Hypothetical proteins | bacterial SH3 domain protein | HP1250 | 77.58 | - | - | - | - | - |
| Hypothetical proteins | Excinuclease ATPase subunit | HP0852 | 83.85 | - | - | - | - | - |
| Hypothetical proteins | NADH-ubiquinone oxidoreductase chain F | HP1265 | 88.66 | 0.018 | 0.040 | 0.078 | 0.190 | 0.214 |
|  |  |  |  |  |  |  |  |  |
| **Control group** |  |  |  |  |  |  |  |  |
| Energy metabolism | ATP synthase F0F1 subunit alpha (atpA) | HP1134 | 98.00 | 0.003 | 0.002 | 0.074 | 0.074 | 0.022 |
| Protein synthesis | elongation factor P (efp) | HP0177 | 98.00 | 0.002 | 0.003 | 0.090 | 0.163 | 0.019 |
| DNA metabolism | A/G-specific adenine glycosylase (mutY) | HP0142 | 94.00 | 0.011 | 0.025 | 0.095 | 0.225 | 0.111 |
| Central intermediary metabolism | inorganic pyrophosphatase (ppa) | HP0620 | 96.00 | 0.004 | 0.004 | 0.060 | 0.119 | 0.102 |
| Tryptophan biosynthesis | anthranilate isomerase (trpC) | HP1279 | 94.00 | 0.020 | 0.032 | 0.088 | 0.188 | 0.168 |
| Central intermediary metabolism | urease accessory protein (ureI) | HP0071 | 97.00 | 0.001 | 0.010 | 0.047 | 0.067 | 0.072 |
| Unknown function | GTP-binding protein (yphC) | HP0834 | 96.00 | 0.008 | 0.018 | 0.078 | 0.154 | 0.097 |

________________________________________________________________________________________________________________

^a^ Outlier sequences were removed prior to these analyses.

^b^ The mean % amino acid identity when comparing East Asian (EA) and non-EA sequences was significantly higher for the control group of housekeeping genes than for

the group of divergent genes

^c^ The mean Ka/Ks ratio, calculated based on comparison of East Asian (EA) sequences with non-EA (NEA) sequences, was significantly higher for the group of divergent genes than

for the control group of housekeeping genes.

^d^ Not applicable. HomB is absent from strain 26695.
