# Supplementary material for: Comparative Genomic Analysis of East Asian and Non-Asian Helicobacter pylori Strains Identifies Rapidly Evolving Genes
Source: PLoS One. 2013 Jan 31;8(1):e55120. doi: 10.1371/journal.pone.0055120 (PMC3561388; doi:10.1371/journal.pone.0055120)
Supplement: Table S2 — Analysis of positive selection using McDonald-Kreitman test (outliers removed). (DOCX) [file pone.0055120.s003.docx]

Table S2. Analysis of positive selection using McDonald-Kreitman test (outliers removed) ^a^

| **Main role** | **Annotation** | **Gene ID (26695)** | ***Dn*** | ***Ds*** | ***Pn*** | ***Ps*** | ***P* value** | **NI** ^b^ | **α-Value^c^** |
| --- | --- | --- | --- | --- | --- | --- | --- | --- | --- |
| Cell envelope | outer membrane protein HopZ (omp1) | HP0009 | 76.77 | 82.44 | 192 | 249 | 0.308 | 0.828 | 0.171 |
| Cell envelope | outer membrane protein HopD (omp2) | HP0025 | 53.18 | 30.30 | 81 | 203 | 0.000 | 0.227* | 0.772 |
| Cell envelope | outer membrane protein BabA (omp28) | HP1243 | - | - | - | - | - | - | - |
| Cell envelope | outer membrane protein HomC/HomD | HP0373 | 155.81 | 96.67 | 94 | 177 | 0.000 | 0.329* | 0.670 |
| Cell envelope | outer membrane protein HomB | NA^d^ | 29.38 | 19.58 | 159 | 280 | 0.001 | 0.378* | 0.621 |
| Cell envelope | outer membrane protein SabA/HopP/(omp17) | HP0725 | 21.21 | 16.44 | 170 | 212 | 0.164 | 0.621 | 0.378 |
| Cell envelope | outer membrane protein HopK (omp12) | HP0923 | - | - | - | - | - | - | - |
| Cell envelope | outer membrane protein HopA (omp6) | HP0229 | 5.01 | 12.32 | 120 | 106 | 0.052 | 2.781 | -1.781 |
| Cell envelope | outer membrane protein HopL (omp26) | HP1157 | - | - | - | - | - | - | - |
| Cell envelope | vacuolating cytotoxin (VacA)-like protein | HP0609/0610 | - | - | - | - | - | - | - |
| Cell envelope | vacuolating cytotoxin (VacA)-like protein | HP0922 | - | - | - | - | - | - | - |
| Cell envelope | hpaA-like protein | HP0492 | - | - | - | - | - | - | - |
| Cell envelope | alpha-(1,3)-fucosyltransferase | HP0651 | - | - | - | - | - | - | - |
| Cell envelope | lipopolysaccharide 1,2-glucosyltransferase (rfaJ) | HP0159 | - | - | - | - | - | - | - |
| Cell envelope | cysteine-rich protein D/beta-lactamase HcpD | HP0160 | 10.09 | 4.05 | 57 | 67 | 0.070 | 0.341 | 0.658 |
| Cellular processes | cytotoxin associated protein A (cagA) | HP0547 | 151.61 | 91.59 | 271 | 190 | 0.360 | 0.861 | 0.138 |
| Cellular processes | vacuolating cytotoxin A | HP0887 | 56.72 | 34.93 | 248 | 397 | 0.000 | 0.384* | 0.615 |
| Cellular processes | Flagellar hook-length control protein | HP0906 | - | - | - | - | - | - | - |
| DNA metabolism | recombination protein RecB/helicase | HP1553 | - | - | - | - | - | - | - |
| DNA metabolism | ribonuclease H (rnhA) | HP0661 | 5.07 | 4.19 | 13 | 20 | 0.403 | 0.537 | 0.462 |
| DNA metabolism | ribonuclease HII (rnhB) | HP1323 | - | - | - | - | - | - | - |
| DNA metabolism | type I restriction enzyme M protein (HsdM) | HP0463 | - | - | - | - | - | - | - |
| DNA metabolism | Type I restriction enzyme M protein (HsdM) | HP0850 | 16.14 | 12.31 | 102 | 154 | 0.082 | 0.505 | 0.494 |
| DNA metabolism | Type IIG restriction-modification enzyme | HP1354 | 11.04 | 9.11 | 377 | 343 | 0.829 | 0.906 | 0.093 |
| DNA metabolism | type III restriction enzyme R protein | HP1371 | - | - | - | - | - | - | - |
| Protein fate | metalloprotease | HP0806 | 8.09 | 4.08 | 41 | 50 | 0.160 | 0.414 | 0.585 |
| Protein fate | preprotein translocase subunit SecG | HP1255 | - | - | - | - | - | - | - |
| Protein synthesis | tRNA delta(2)-isopentenylpyrophosphate transferase (miaA) | HP1415 | 12.15 | 8.26 | 81 | 90 | 0.298 | 0.611 | 0.388 |
| Protein synthesis | selenocysteine synthase (SelA)/L-seryl-tRNA(Sec) selenium transferase | HP1513 | 16.20 | 14.59 | 75 | 123 | 0.120 | 0.549 | 0.450 |
| Purines, pyrimidines, nucleosides, and nucleotides | purine nucleoside phosphorylase (punB) | HP1530 | 7.07 | 2.02 | 41 | 53 | 0.048 | 0.221* | 0.778 |
| Transcription | poly(A) polymerase (papS) | HP0640 | 9.06 | 11.35 | 49 | 84 | 0.512 | 0.730 | 0.269 |
| Unknown function | poly E-rich protein | HP0322 | 5.01 | 4.05 | 116 | 107 | 0.845 | 0.875 | 0.124 |
| Hypothetical proteins | tRNA(Ile)-lysidine synthase | HP0728 | 9.06 | 8.21 | 48 | 52 | 0.731 | 0.836 | 0.163 |
| Hypothetical proteins | probable ATP /GTP binding protein | HP0729 | - | - | - | - | - | - | - |
| Hypothetical proteins | bacterial SH3 domain protein | HP1250 | - | - | - | - | - | - | - |
| Hypothetical proteins | Excinuclease ATPase subunit | HP0852 | - | - | - | - | - | - | - |
| Hypothetical proteins | NADH-ubiquinone oxidoreductase chain F | HP1265 | 7.05 | 3.03 | 53 | 57 | 0.186 | 0.400 | 0.599 |
|  |  |  |  |  |  |  |  |  |  |
| **Control group** |  |  |  |  |  |  |  |  |  |
| Energy metabolism | ATP synthase F0F1 subunit alpha (atpA) | HP1134 | 0.00 | 4.03 | 5 | 73 | 0.599 | Null | Null |
| Protein synthesis | elongation factor P (efp) | HP0177 | 0.00 | 3.05 | 4 | 49 | 0.618 | Null | Null |
| DNA metabolism | A/G-specific adenine glycosylase (mutY) | HP0142 | 1.00 | 4.06 | 29 | 76 | 0.698 | 1.547 | -0.547 |
| Central intermediary metabolism | inorganic pyrophosphatase (ppa) | HP0620 | 5.04 | 5.16 | 1 | 20 | 0.003 | 0.051* | 0.948 |
| Tryptophan biosynthesis | anthranilate isomerase (trpC) | HP1279 | 7.03 | 12.34 | 54 | 100 | 0.915 | 0.948 | 0.051 |
| Central intermediary metabolism | urease accessory protein (ureI) | HP0071 | 0.00 | 4.08 | 10 | 35 | 0.285 | Null | Null |
| Unknown function | GTP-binding protein (yphC) | HP0834 | 3.01 | 6.14 | 15 | 47 | 0.573 | 0.652 | 0.347 |

________________________________________________________________________________________________________

^a^ Outlier sequences were removed prior to these analyses.

^b^ The neutrality index (NI) was calculated from the ratio of the number of polymorphisms to the number of substitutions as follows: NI = (*Pn*/*Ps*)/(*D*n/*Ds*), where *P* is

polymorphic within the population, *D* is divergence or fixed difference between populations, *n* is nonsynonymous, and *s* is synonymous.

^c^ The proportion of adaptive substitutions that ranges from - ∞ to 1 and is estimated as 1 - NI.

^d^ Not applicable. HomB is absent from strain 26695.

*Asterisks indicate genes showing signatures of diversifying selection.
